# Identification of suicidal ideation in major depressive disorder: A machine learning approach with multimodal digital features

**DOI:** 10.1371/journal.pdig.0001615

**Published:** 2026-08-27

**Authors:** Marco Shing Yan Man, Andy Lok Man Au, Christopher Chi Wai Cheng, Mavis Ka Yan Siew, Yuyun Liu, Kit Ying Chan, Jie Chen, Joey Wing Yan Chan, Yun Kwok Wing, Tim Man Ho Li

**Affiliations:** 1 Faculty of Medicine, The Chinese University of Hong Kong, Hong Kong, China; 2 Li Chiu Kong Family Sleep Assessment Unit, Department of Psychiatry, The Chinese University of Hong Kong, Hong Kong, China; 3 Li Ka Shing Institute of Health Sciences, Faculty of Medicine, The Chinese University of Hong Kong, Hong Kong, China; 4 Department of Psychiatry, Fujian Medical University Affiliated Fuzhou Neuropsychiatric Hospital, Fuzhou, China; 5 Gerald Choa Neuroscience Institute, The Chinese University of Hong Kong, Hong Kong, China; Army Medical University, CHINA

## Abstract

Current detection models for suicidal ideation (SI) among depressed patients have primarily relied on clinical and biological features. This study aims at enhancing the real-world applicability of these models by using multimodal digital features. Patients self-administered the Hospital Anxiety and Depression Scale (HADS) and 20-item Toronto Alexithymia Scale (TAS-20). Their multimodal features (facial, acoustic and linguistic features) were obtained using ecological momentary assessment (EMA) with our self-developed smartphone application over 7 days. The Structured Interview Guide for the Hamilton Depression Rating Scale (HDRS) were conducted to identify clinically rated SI. Mood description recordings obtained from EMA were processed through natural language processing, Openface, and OpenSmile to obtain linguistic, facial and acoustics features, respectively. Multimodal features and questionnaires were then used for training and testing machine learning algorithms. The study was conducted and reported under the TRIPOD+AI guideline. Among the recruited 99 patients with major depressive disorder (MDD), 38 (mean age = 49.3 ± 9.7 y, 76% female) had SI while 61 (mean age = 51.5 ± 11.5 y, 76% female) did not have SI. Among the 10 machine learning models evaluated, the CatBoost classifier demonstrated the strongest detection performance, achieving an AUC of 0.79 (p < .001), accuracy of 0.79 and F1-score of 0.73. K-Nearest Neighbours (KNN), Artificial Neural Network (ANN) and Naive Bayes models also provided comparable results. Multimodal features were correlated with SI among MDD patients. Machine learning algorithms have modest performance in identifying SI among patients with MDD using multimodal digital features, including facial expressions, vocal characteristics and language use. However, larger and more diverse datasets are needed to enhance the generalizability and accuracy of these machine learning approaches in real-world clinical settings.

## Author summary

Suicide remains a critical public health challenge, particularly among people living with depression. Traditional methods of detecting suicidal thoughts rely on clinical interviews, which limits our ability to identify at-risk individuals outside hospital settings. While recent machine learning approaches show promising result, most require specialized medical data like brain scans or electronic health records that are only available in clinical environments. Suicidal ideation (SI) beyond hospital field is thus often out of detection range and could not be reached. In this study, we utilized a self-developed mobile app to collect data, allowing patients to conduct ecological momentary assessment and questionnaires without professional assistant. Videos and questionnaire data collected remotely were then processed through natural language processing and more to identify SI within patients. We found this approach shows potential results detecting suicide risk in people with depression, demonstrating feasibility in community-based screening. This mobile-based method could extend suicide prevention beyond hospital walls, reaching individuals in their daily lives when early intervention matters most, moving towards a more proactive suicide prevention strategy.

## Introduction

Suicide is a leading cause of death, contributing to the death of more than 700,000 people every year [1]. While suicide is a multifactorial phenomenon, having a mental disorder is a major risk factor, with 90% of people who commit suicide having a history of mental disorder [2]. Major depressive disorder (MDD) is a common mental disorder, which is strongly associated with suicidal outcomes [3], and accounts for the majority of suicidal cases related to mental disorder [2].

Identifying individuals at risk for suicide among the MDD population often represents a significant clinical challenge. Assessment strategies that are currently adopted largely depend on subjective self-report measures [4]. However, there are growing concerns about the reliability and accuracy of these methods, as people may struggle to accurately express or even recognize their feelings and suicidal thoughts (for example, those with alexithymia), making it difficult to capture the true extent of suicidality through self-reports alone [5]. Patients may not accurately recall, or be able to articulate their emotional states and suicidal thoughts [6]. While clinician-administered assessments may provide greater accuracy by reviewing patients’ medical histories and asking systematic questions to clarify their thoughts, the waiting times for clinical appointment, and a shortage of trained healthcare professionals limit their scalability and hinder the timely identification of those at risk for suicide.

Ecological momentary assessment (EMA) has been used to collect real-life data from patients [7]. EMA gathers real-time data, minimizing recall bias and yielding more precise information compared to retrospective questionnaires. There is growing interest in integrating additional modalities, including facial expressions, vocal characteristics and language use, into EMA to collect more comprehensive data for analysis [8,9]. This approach aims to enhance the richness and depth of the information gathered through EMA. Evidence from studies in other mental disorders further suggests that these multimodal approaches may be valuable for detection and clinical risk prediction [10,11]. Despite these advancements, multimodal analysis for suicidal ideation (SI) detection remains limited. A recent review found that only 3 out of 14 multimodal, speech-based studies focused specifically on SI detection [12]. While suicidal ideation typically occurs before suicidal behaviors, these methods have the potential to identify risk at an earlier stage, enhancing suicide prevention efforts.

Recent machine learning studies have demonstrated the potential of diverse data modalities for suicide-related risk stratification in MDD and other psychiatric populations [13]. Whole-brain functional connectivity has been used to distinguish patients with high versus low suicide risk [14], while dynamic functional connectivity and connectomics-based resting-state network analyses have further suggested that suicidality is associated with disruption of functional brain organization [15,16]. Other studies have shown that integrating pain measures with event-related potential features [17] or combining neuroimaging with clinical text [18] may improve the classification of suicide attempt or suicidal ideation in MDD. In addition, hospital-based models using demographic, clinical, and biological variables have identified correlates of suicidal ideation in MDD [19], and natural language processing of unstructured clinical notes has provided incremental predictive value beyond electronic health record variables in suicide prediction models [20]. However, many existing models rely on resource-intensive or difficult-to-scale data sources, and relatively little is known about whether real-life, multimodal EMA-derived features could improve the identification of suicidal ideation in individuals with MDD.

This study examines the utility of a multimodal EMA-machine learning framework for the identification of clinically rated SI in individuals diagnosed with MDD, with the objective of contributing to more accurate risk stratification, assessing incremental value of multimodal features and facilitating timely intervention in real-world settings.

## Methods

### Ethical statement

All procedures conformed to ethical standards outlined in the Declaration of Helsinki. Ethics approval was granted by the Joint Chinese University of Hong Kong—New Territories East Cluster Clinical Research Ethics Committee (Ref No: 2020.492). Before enrolment, each participant was provided with a detailed explanation of the study objectives and methods, after which informed written consent was obtained.

### Study design

This research is an on-going study aimed at developing a digital mental health platform for diagnosing and assessing MDD subjects [8,9]. This study presents an interim analysis of the data collected between June 2021 and January 2025.

### Study population

The study population consisted of adults aged 18–65 years. Individuals diagnosed with MDD were primarily recruited from a regional psychiatric outpatient clinic in Hong Kong. Their diagnoses were established by attending psychiatrists through clinical evaluation. In parallel, approximately one-fifth of the MDD cohort was identified via community outreach. These community participants were assessed through the Structured Clinical Interview for DSM-5 Clinician Version (SCID-5-CV), administered by a trained medical researcher to ensure diagnostic accuracy.

Participants were excluded if they met any of the following criteria: a lifetime history of bipolar disorder, schizophrenia-spectrum disorders, neurodegenerative, neurodevelopmental or neurological illnesses. Additional exclusion criteria included conditions or circumstances that could affect data quality or confound results, such as facial trauma, speech impediments, employment involving night shifts, or physical motor impairments [8,9,21].

### Clinical assessment

The Structured Interview Guide for the Hamilton Depression Rating Scale (HDRS) was conducted to assess the depressive symptoms by a trained medical researcher [22]. To specifically assess SI, the item H11 of the HDRS was utilized, with all remaining items excluded to mitigate the risk of data leakage for the interview data, patients with rating 1 or above in the item were grouped to facilitate classification tasks. H11 asks “Since last week, have you had any thoughts that life is not worth living?” Suicide risk was rated in five progressive levels: (0) having no suicidal thoughts; (1) feeling life is not worth living; (2) having wishes to be dead, or any thoughts of possible death of self; (3) having SI or gestures; and (4) having attempts at suicide.

The Hospital Anxiety and Depression Scale (HADS) [23] was used for assessing depressive (HADS-D) and anxiety symptoms (HADS-A). Subjects also completed a 20-item Toronto Alexithymia Scale (TAS-20) to assess alexithymia, obtaining Toronto Alexithymia Scale-Difficulty Identifying Feelings score (TAS-DIF) and Toronto Alexithymia Scale-Difficulty Describing Feelings(TAS-DDF) score [24].

### Multimodal ecological momentary assessment

Participants completed EMA using our self-developed smartphone application (D-MOMO) for 7 days [9]. Each participant recorded their real-time mood states four times per day over seven consecutive days. The EMA were audio-video-based, with both the camera and microphone activated, and participants were prompted to answer two questions: (1) their current mood and (2) activities undertaken since the previous entry. These recordings captured multimodal features including facial expressions, acoustic properties, and verbal content. Analysis of facial, vocal, and language features are established to show their feasibility for identifying psychiatric disorders [8,9].

### Feature extraction

Facial behavior analysis was performed using OpenFace 2.2.0 [25], a state-of-the-art computer vision toolkit for facial action unit (AU) recognition, according to the Facial Action Coding System. The system tracked a predefined set of AUs at 26 frames per second, generating frame-wise facial features. To ensure data quality, based on a preliminary sensitivity analysis, a confidence threshold of 0.7 was applied, retaining only frames above the threshold. Video-level features were then derived by aggregating frame-level values (e.g., mean AU counts). In addition, peak AU intensities and change velocities were computed to capture dynamic information. The facial feature pipeline yielded 36 features, including indicators of video quality such as the mean AU detection confidence.

Speech segments were extracted from the mood diary recordings for speech and language processing. Automatic speech recognition (ASR) was performed using Tencent Cloud, with transcripts verified against manually transcribed ground truth by native Cantonese-speaking research staff. Acoustic features were extracted with Praat [26] and included fundamental frequency (F0) mean and variability, articulation rate, mean and variability of pause duration, and pause rate. To extend the acoustic representation, low-level descriptors (e.g., mel-frequency cepstral coefficients) were extracted using OpenSMILE [27]. To reduce environmental interference, human vocal signals were isolated using a U-Net convolutional architecture implemented in Demucs for music source separation [28]. In total, 84 acoustic features were obtained.

Transcripts were processed using fastHan 1.8.0 [29], a deep learning-based Chinese word segmentation engine. Segmented words were then categorized into 79 linguistically and psychologically meaningful categories based on the Cantonese version of the Language Inquiry and Word Count (LIWC) dictionary [30]. Together with the total word count, this yielded 80 linguistic features.

All features were summarized using both static and dynamic measures. Static measures included means, while dynamic measures included standard deviation, root mean square of successive differences, within-day changes, and between-day changes. In total, 980 derived features were generated. With the addition of demographic information (e.g., gender, age, and education level) and questionnaires (TAS, HADS), the final dataset comprised 1009 features for further machine learning analyses.

### Machine learning algorithms

All machine learning analyses were performed using Python (version 3.13). Preprocessing procedures were implemented in pipeline to optimise subsequent machine learning outcomes. Missing data (0.05%), limited to demographic and questionnaire information, were addressed using K-Nearest Neighbours (KNN) imputation. Furthermore, features were standardised using the “StandardScaler” [31] from scikit-learn to ensure appropriate scaling for models sensitive to feature magnitudes. Since only mild imbalance was present in the dataset, Synthetic Minority Oversampling Technique (SMOTE) was not used to address class imbalance in the training set.

To assess the detection capability for identifying individuals at risk of SI, ten machine learning models were evaluated using the dataset through internal validation using all the above-mentioned data except HDRS data. Hyperparameter grid search and five-fold cross-validation were employed to enhance the generalizability of models, the full specification and results of the grid search are in the supplementary material (S1 Appendix). To reduce data dimensionality, minimise noise and improve model performance [32], feature selection was performed within each cross-validation fold and hyperparameter setting to prevent data leakage by calculating the ANOVA F-value during training. We incrementally selected features ranging from n = 10 up to all features, (n = 10, 20, 30, 40, 50, 100, 500, 1009), to select the best number of features suitable for each model (detail in S1 Appendix). Model selection was mainly based on the F1-score and AUC, thresholds are calibrated using an F1-prioritized criterion. This is considered more suitable for clinical decision-making contexts and binary classification tasks, especially under the mild imbalanced state of the data [33]. Precision-recall statistics were preferred over ROC curves, offering more informative visual cues and greater accuracy in the context of imbalanced datasets [34].

All models underwent internal validation, with performance evaluated using the area under the receiver operating characteristic curve (AUC), accuracy, F1-score, sensitivity, specificity, positive predictive value (PPV) and negative predictive value (NPV). The AUC value for machine learning algorithms was evaluated through the cross-validation AUC due to the limited size of the dataset, to reduce the variance of the accuracy estimate [35].

This study was conducted in accordance with the Transparent Reporting of a multivariable prediction model for Individual Prognosis Or Diagnosis + artificial intelligence TRIPOD+AI guideline [36] to promote transparency in model development and minimise potential biases.

### Statistical analysis

All processed data were analysed utilising R software (R version 4.4.2). Descriptive statistics were presented as mean (SD) or frequencies (%). The two-sample t-test is employed to examine the differences in means of normally distributed continuous variables between the MDD subjects with and without SI. P-values are presented for exploratory and descriptive purposes rather than strict hypothesis testing. Concurrently, Fisher’s exact test is applied to assess the intergroup differences of categorical variables. A p-value of less than 0.05 was considered statistically significant. All hypothesis tests were two-sided.

## Results

### Participants’ characteristics

A total of 99 individuals diagnosed with MDD (n = 99) were assessed with the H11 item. 61 individuals were identified as non-suicidal MDD. In contrast, 38 individuals were categorised as MDD patients exhibiting SI. Table 1 presents a summary of demographic data. There were no notable intergroup differences in age (p = .80), sex (p > .99), or other factors. Nevertheless, a significantly higher rate of unmarried individuals was in SI subjects (29.5% vs 55.3%, p = .01).

**Table 1:** Demographic Information between MDD subjects without SI and MDD subjects with SI.

	MDD without SI (n = 61)	MDD with SI (n = 38)
**Age, mean (SD)**	51.5 (11.5)	49.3 (9.7)
**Female, N (%)**	47 (77.0)	29 (76.3)
**Married, N (%)**	43 (70.5)	17 (44.7)
**Secondary school or above, %**	96.7	94.7
**Employment status, yes, %**	50.8	55.3
**Family income monthly <15,000 HKD, %**	29.5	47.4

p-value on independent sample t-test or chi-squared test.

Table 2 summarises the results of clinical characteristics. HADS-A (7.30 vs. 11.00, p < .001) and HADS-D (6.95 vs. 11.55, p < .001) scores were significantly higher in the SI group than the non-suicidal group. Similarly, TAS-DIF (17.18 vs 21.76, p = .002) and TAS-DDF (13.25 vs 15.16, p = .01) were significantly higher in the SI group, indicating difficulty in identifying feelings and difficulty describing feelings. Nevertheless, the capability for externally oriented thinking did not show a significant difference between the two groups.

**Table 2:** Clinical assessment data comparison between MDD subjects without SI and MDD subjects with SI, significant features were reported.

	MDD without SI (n = 61)	MDD with SI (n = 38)
**HADS-A, mean (SD)**	7.30 (3.97)	11.00 (4.56)
**HADS-D, mean (SD)**	6.95 (4.07)	11.55 (4.16)
**TAS-DIF, mean (SD)**	17.18 (6.32)	21.76 (7.17)
**TAS-DDF, mean (SD)**	13.25 (3.51)	15.16 (3.60)
**TAS-EOT, mean (SD)**	25.90 (4.83)	24.55 (5.48)

Table 3 displays the outcomes of the acquired facial features. Among the collected AUs, the mean (17.28 vs 10.04, p = .03) and the within-day change (10.17 vs 6.18, p = .002) of lip corner pulling (AU12) indicated a significant difference between the non-suicidal and SI subjects. Other facial features did not exhibit any significant intergroup differences.

**Table 3:** Multi-modality features between MDD subject without SI and MDD subject with SI.

Facial expression: AU, mean (SD), %	MDD without SI (n = 61)	MDD with SI (n = 38)	p-value
**1 (Inner brow raising)**	22.79 (7.62)	22.66 (5.19)	.92
**4 (Brow lowering)**	29.15 (20.52)	32.08 (26.48)	.56
**6 (Cheek raising)**	22.90 (22.04)	20.16 (16.34)	.48
**12 (Lip corner pulling)**	17.28 (17.34)	10.44 (14.06)	.03*
**15 (Lip corner depressing)**	20.72 (9.86)	21.45 (8.11)	.69
**17 (Chin Raiser)**	27.74 (8.58)	27.17 (6.81)	.72
**25 (Lips part)**	29.60 (6.62)	28.80 (5.77)	.53
**Facial expression: AU, within day change (SD), %**			
**1 (Inner brow raising)**	7.06 (2.43)	7.16 (2.51)	.85
**4 (Brow lowering)**	15.20 (7.79)	12.64 (6.63)	.08
**6 (Cheek raising)**	11.51 (7.61)	11.80 (6.56)	.84
**12 (Lip corner pulling)**	10.17 (7.17)	6.18 (5.10)	.002**
**15 (Lip corner depressing)**	7.84 (2.54)	8.28 (2.48)	.50
**17 (Chin Raiser)**	8.49 (2.29)	8.64 (3.45)	.81
**25 (Lips part)**	5.70 (1.96)	6.07 (2.38)	.42

p-value * < .05, ** < .01.

Table 4 displays the outcomes of the acquired linguistic and acoustic features, a higher frequency of self-referencing in the SI group than the non-suicidal group is shown (1.54 vs 1.06, p = .03). Nevertheless, there was no significant inter-group difference in negative emotion. Acoustic Feature analysis revealed several significant differences between the two groups. Mel-Frequency Cepstral Coefficients (MFCC), which captures tone, pitch, voice energy and clarity, has also shown significant differences. MFCC 1 (27.50 v 29.32, p = .04) and MFCC 3 (3.64 vs 6.76, p = .04) were significantly higher in SI group. F0 Frequency, variability and Articulation rate were lower in a group with SI but did not reach the level of significance.

**Table 4:** Acoustic and Linguistic features between MDD subject without SI and MDD subject with SI.

Linguistic Feature, mean (SD)	MDD without SI (n = 61)	MDD with SI (n = 38)	p-value
**Self-reference**	1.54 (1.44)	1.06 (0.68)	.03*
**Anxiety**	0.34 (0.60)	0.34 (0.51)	>.99
**Anger**	0.23 (0.66)	0.23 (0.47)	>.99
**Sad**	0.09 (0.12)	0.26 (0.62)	.10
**Positive motion**	4.40 (1.94)	5.01 (2.23)	.17
**Negative motion**	0.98 (1.04)	1.33 (1.25)	.15
**Female**	0.21 (0.36)	0.10 (0.21)	.05*
**Reward**	1.70 (0.83)	2.53 (1.68)	.007**
**Focus_past**	0.33 (0.55)	0.48 (0.60)	.22
**Focus_present**	0.80 (0.63)	1.05 (0.75)	.10
**Focus_future**	1.02 (0.79)	0.86 (0.80)	.33
**Acoustic Feature, mean (SD)**			
**F0 Mean (Hz)**	164.485 (31.852)	153.90 (23.30)	.06
**F0 Variability (Hz)**	53.69 (14.31)	48.73 (11.47)	.06
**Articulation Rate**	6.12 (6.35)	4.62 (2.84)	.11
**F0 Frequency**	28.92 (3.60)	28.03 (2.44)	.15
**Range of F0**	8.49 (2.50)	7.86 (1.78)	.15
**Loudness**	0.80 (0.31)	0.76 (0.36)	.62
**Spectral Reflux**	0.59 (0.34)	0.55 (0.46)	.61
**MFCC1**	27.50 (4.50)	29.32 (3.85)	.04*
**MFCC2**	5.63 (5.05)	6.65 (5.49)	.36
**MFCC3**	3.64 (6.60)	6.76 (7.44)	.04*
**F1 Frequency**	579.61 (47.12)	571.37 (31.66)	.30
**F2 Frequency**	1580.41 (73.92)	1576.63 (41.27)	.75
**F3 Frequency**	2633.42 (95.42)	2638.87 (55.01)	.72

p-value * < .05, ** < .01.

### Machine learning outcomes

The detection performance of 10 different machine learning models was systematically assessed for the identification of the SI group among the MDD patients (Table 5). Among the 10 machine learning models evaluated, the CatBoost classifier showed the best overall performance, achieving an AUC of 0.79 (95% CI: 0.69–0.89), an F1-score of 0.73 (95% CI: 0.61–0.83), an accuracy of 0.79, a sensitivity of 0.76, and a specificity of 0.80. It also yielded a PPV of 0.71 and an NPV of 0.84, indicating the most balanced performance among the evaluated models. Several other models also showed comparable performance. K-nearest neighbors (KNN) demonstrated comparatively strong performance, with an AUC of 0.79 (95% CI: 0.69–0.87), an F1-score of 0.71 (95% CI: 0.59–0.81), an accuracy of 0.77, a sensitivity of 0.74, and a specificity of 0.79. Naive Bayes (NB) likewise showed competitive performance, with an AUC of 0.72 (95% CI: 0.62–0.81), an F1-score of 0.58 (95% CI: 0.44–0.70), an accuracy of 0.66, a sensitivity of 0.61, and a specificity of 0.69. Artificial neural network (ANN) showed moderate discrimination, with an AUC of 0.73 (95% CI: 0.62–0.82) and an F1-score of 0.64 (95% CI: 0.51–0.74), whereas logistic regression achieved high sensitivity (0.92) but lower specificity (0.38). For decision tree and random forest models, they both showed high sensitivity but reduced specificity. Gradient boost and XGBoost showed lower F1-score and AUC when compared to other models.

**Table 5:** Performance metrics for internal validation of evaluated machine learning models for the detection of SI within MDD patients (n = 99).

Model	AUC (95% CI)	F1 Score (95% CI)	Accuracy	Sensitivity		Specificity	PPV	NPV
**ANN**	0.73 (0.62–0.82)	0.64 (0.51–0.74)	0.69	0.71		0.67	0.57	0.79
**Catboost**	0.79 (0.69–0.89)	0.73 (0.61–0.83)	0.79	0.76		0.80	0.71	0.84
**DT**	0.69 (0.58–0.79)	0.62 (0.51–0.71)	0.60	0.84		0.44	0.48	0.82
**GB**	0.61 (0.49–0.73)	0.48 (0.34–0.63)	0.66	0.42		0.80	0.57	0.69
**KNN**	0.79 (0.69, 0.87)	0.71 (0.59–0.81)	0.77	0.74		0.79	0.68	0.83
**LR**	0.70 (0.59–0.79)	0.63 (0.52–0.73)	0.59	0.92		0.38	0.48	0.88
**NB**	0.72 (0.62–0.81)	0.58 (0.44–0.70)	0.66	0.61		0.69	0.55	0.74
**RF**	0.68 (0.56–0.80)	0.56 (0.44–0.66)	0.51	0.82		0.31	0.42	0.73
**SVM**	0.67 (0.56–0.78)	0.58 (0.45–0.70)	0.63	0.68		0.59	0.51	0.75
**XG**	0.59 (0.47-0.71)	0.49(0.33-0.63)	0.66	0.42		0.80	0.57	0.69

Abbreviations: AUC = Area under the receiver operating characteristic curve; ANN = Artificial Neural Network, DT = Decision Tree, GB = Gradient Boost, KNN = k-Nearest Neighbors; LR = Logistic Regression, NB = Naive Bayes, RF = Random Forest, SVM = Support Vector Machine, CatBoost = Categorical Boosting, XGBoost = Extreme Gradient Boosting.

Overall, CatBoost provided the most favorable trade-off across AUC, F1-score, sensitivity, specificity, PPV, and NPV, while KNN, Naive Bayes and ANN also demonstrated a reasonably balanced performance profile, indicating potential for further optimization. These findings suggest that multimodal digital features could potentially have incremental value for models in identifying individuals at elevated risk of suicide among patients with MDD.

### Feature importance

**Fig 1:**
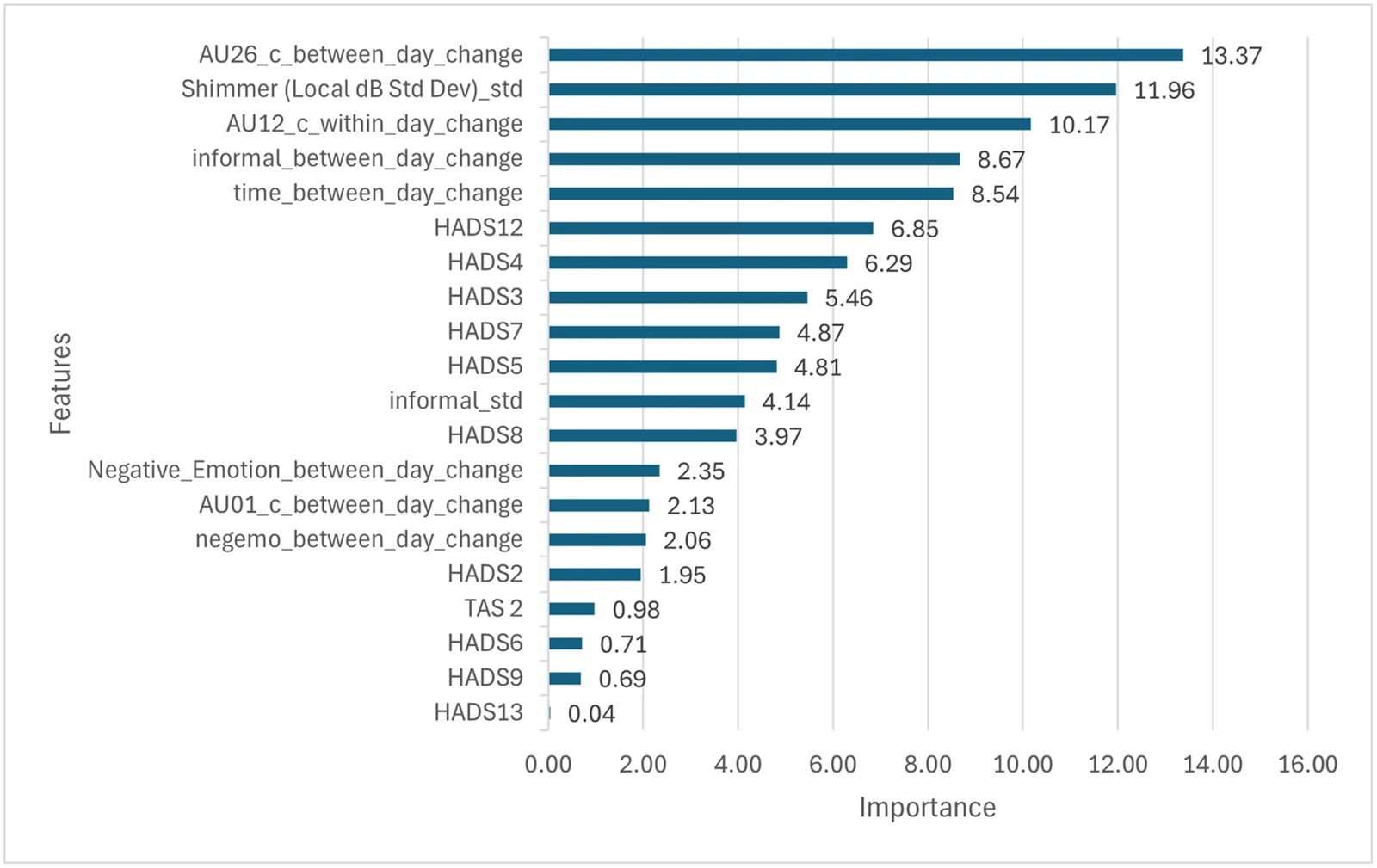
Feature importance for best CatBoost model. Abbreviations: HADS = Hospital Anxiety and Depression Scale, AU = facial Action Unit, TAS = Toronto Alexithymia Scale, Shimmer (Local dB Std Dev)_std denotes the standard deviation of local shimmer in dB. Informal, Negative_Emotion, negemo, and time denote linguistic feature categories. Std = standard deviation, features with _between_day_change indicate day-to-day variability across the assessment period, features with _within_day_change indicate within-day fluctuation.

Based on the feature importance of the best performing models, the predictive information was distributed across multiple modalities. In the CatBoost model, the strongest predictors included facial, acoustic, linguistic variables, and several HADS items, with AU26, Shimmer, and AU12 ranking highest (see Fig 1).

**Fig 2:**
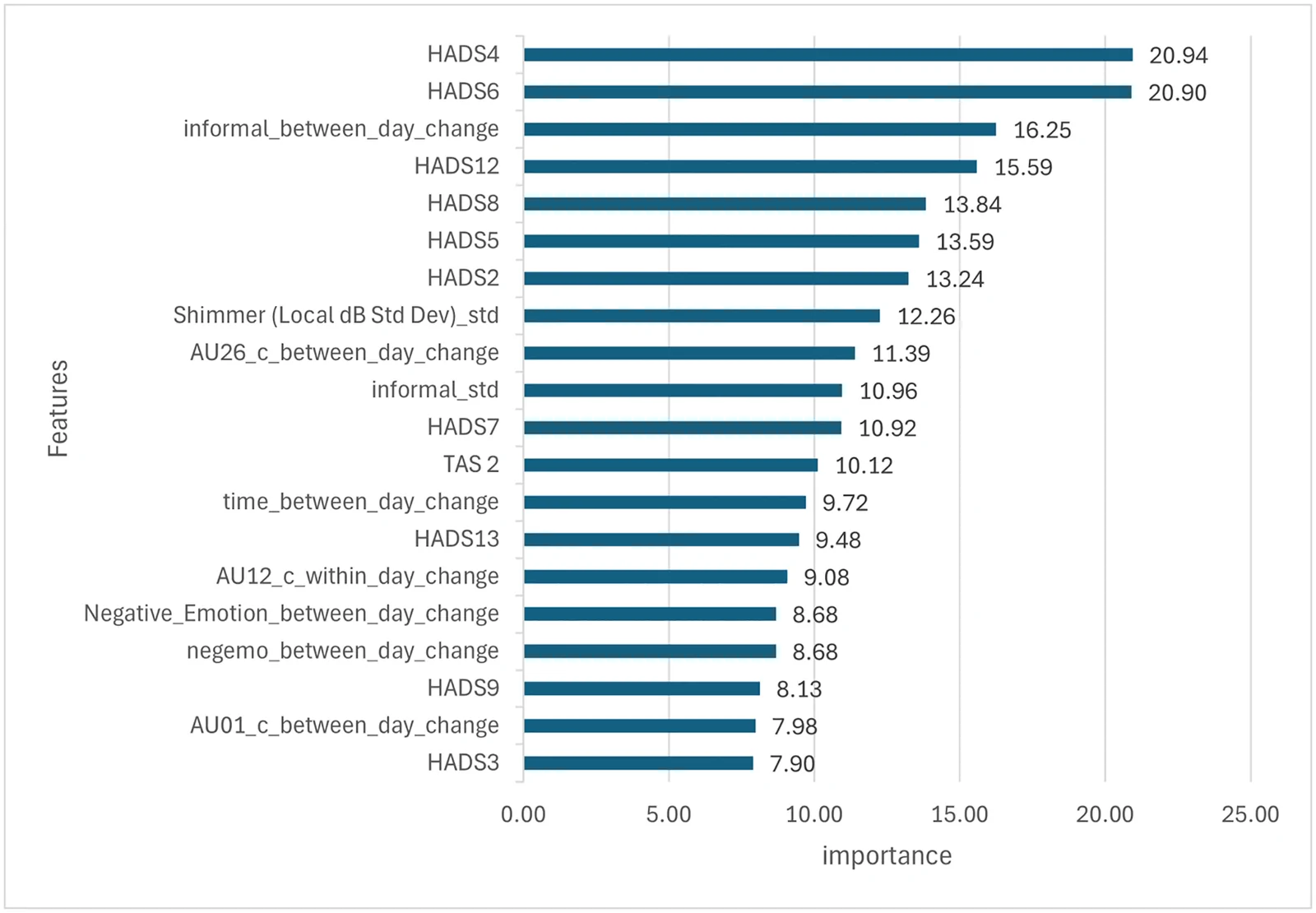
Feature importance for best naïve bayes model. Abbreviations: HADS = Hospital Anxiety and Depression Scale, AU = facial Action Unit, TAS = Toronto Alexithymia Scale, Shimmer (Local dB Std Dev)_std denotes the standard deviation of local shimmer in dB. Informal, Negative_Emotion, negemo, and time denote linguistic feature categories. Std = standard deviation, features with _between_day_change indicate day-to-day variability across the assessment period, features with _within_day_change indicate within-day fluctuation.

In the Naive Bayes model, questionnaire variables were relatively more prominent, but multimodal variables also showed relevant importance, including linguistic variable “informal”, Shimmer and AU26 (see Fig 2).

Facial features such as AU12 and AU26, linguistic features including negative emotion, time, and informality, as well as the acoustic variable shimmer, consistently showed relative importance across the models. Greater contribution by facial and linguistic features are seen over acoustic features.

To assess reproducibility, we examined the consistency of multimodal feature selection across the five cross-validation folds. Several features recurred across folds in both models. In CatBoost, linguistic variable “informal” ranked highly in all 5 folds, with AU26 and Shimmer in 4 folds, and AU12 and linguistic variable “time” in 3 folds. The Naïve Bayes model showed a similar pattern, with linguistic variable “informal” selected in all 5 folds, Shimmer in 4 folds, and AU26, AU12, and linguistic variable “time” in 3 folds. The convergence of the same facial (AU12, AU26), acoustic (shimmer), and linguistic (informality, time) features across folds and across two distinct models indicates that these multimodal signals were selected in a stable and reproducible manner. Overall, the findings support a multimodal contribution to prediction, with the noticeable multimodal features.

### Baseline models

To assess whether multimodal digital features added predictive value beyond conventional clinical information, we compared models trained on demographic and questionnaire variables (Table 6) alone with models trained on broader feature sets including multimodal data. The best performance was obtained using the combined demographic, questionnaire, and multimodal with CatBoost achieving an AUC of 0.79, F1 score of 0.73, and accuracy of 0.79. In comparison, the best baseline model using demographic and questionnaire features alone achieved lower performance. The difference between the baseline model and multimodal models is 5–8% in term of AUC and F1-score, greater improvement is seen across accuracy, specificity, PPV and NPV. This pattern indicates that multimodal features provide potential incremental predictive information beyond standard self-report and demographic measures. Importantly, the highest performance was not obtained from multimodal data alone, but from their integration with conventional variables, suggesting that multimodal feature could be useful as an additive to questionnaire-based assessment.

**Table 6:** Performance metrics for internal validation of evaluated machine learning models for the detection of SI within MDD patients using only demographic and questionnaire features (n = 99).

Model	AUC (95% CI)	F1 Score (95% CI)	Accuracy	Sensitivity		Specificity	PPV	NPV
**ANN**	0.70 (0.58, 0.81)	0.59 (0.46, 0.69)	0.59	0.76		0.48	0.48	0.76
**Catboost**	0.72 (0.61, 0.83)	0.62 (0.49, 0.74)	0.65	0.76		0.57	0.53	0.80
**DT**	0.66 (0.55, 0.76)	0.57 (0.43, 0.69)	0.66	0.61		0.69	0.55	0.74
**GB**	0.67 (0.56, 0.78)	0.53 (0.39, 0.66)	0.65	0.53		0.72	0.54	0.71
**KNN**	0.67 (0.56, 0.78)	0.57 (0.44, 0.68)	0.62	0.66		0.59	0.5	0.73
**LR**	0.74 (0.64, 0.83)	0.65 (0.55, 0.75)	0.62	0.95		0.41	0.5	0.93
**NB**	0.64 (0.53, 0.74)	0.53 (0.41, 0.65)	0.58	0.63		0.54	0.46	0.54
**RF**	0.74 (0.62, 0.84)	0.53 (0.39, 0.66)	0.65	0.53		0.72	0.54	0.71
**SVM**	0.63 (0.51, 0.74)	0.53 (0.40, 0.65)	0.6	0.61		0.59	0.48	0.71
**XG**	0.64 (0.52, 0.75)	0.41 (0.24, 0.54)	0.62	0.34		0.79	0.50	0.66

Abbreviations: AUC = Area under the receiver operating characteristic curve; ANN = Artificial Neural Network, DT = Decision Tree, GB = Gradient Boost, KNN = k-Nearest Neighbors; LR = Logistic Regression, NB = Naive Bayes, RF = Random Forest, SVM = Support Vector Machine, CatBoost = Categorical Boosting, XGBoost = Extreme Gradient Boosting.

An additional pattern was observed across model types. Baseline models using demographic and questionnaire features tended to perform relatively well with simpler models such as logistic regression and random forest, whereas models incorporating multimodal features usually achieved stronger performance with more flexible approaches such as ANN and Catboost. Although this pattern should be interpreted cautiously, it may suggest that the relationship between multimodal features and SI is more complex and potentially more nonlinear than questionnaire and demographic features, requiring models that can better capture feature interactions and higher-order structure.

## Discussion

This study is among the first to detect SI using multimodal digital features derived from EMA, self-administered questionnaires, and videos recorded in everyday real-world environments. This work also builds on early precedent showing that videotaped analysis of non-verbal behavior in depressed patients can yield clinically meaningful signals, as demonstrated by Ulrich in 1985 [37]. Cross-validation results suggested potentially useful performance across several machine-learning models., with the AUC and other metrics, such as accuracy and F1-score, indicating satisfactory performance. The results show potential ability to identify individuals at risk for suicide among patients with MDD.

When compared with previous studies attempting to find effective detection model in capturing suicidality within MDD patients, the models in this study demonstrated comparable performance. Prior research has reported model performance with AUC values ranging from approximately 0.70 to 0.85, often utilizing larger sample sizes and clinical and biological features that are less accessible in regular clinical practice such as health records, MRI scans, and biological samples. A large-scale study (n = 1916) utilized demographic, clinical, and biological variables to employ a neural network algorithm which has achieved an AUC of 0.76 in identifying SI among Chinese patients with MDD [19]. Other investigations that combined clinical symptoms with structural brain features, clinical notes, or brain MRI data reported AUCs of 0.79, 0.75, and 0.74 respectively, the fusion model also achieved an F1 score of 0.78 in internal validation [18]. Importantly, these earlier multimodal approaches were generally based on hospital-derived data or clinician guided interview or different assessment protocols, whereas our study collected EMA responses and questionnaires entirely through a mobile application in real-world daily settings, without requiring involvement of synchronous clinician/research personnel during data acquisition, in a more accessible and naturalistic setting. These findings suggest that convenient and continuous multimodal digital data are feasible to collect in real-world settings to allow potential early identification of individuals at risk for suicide among patients with MDD in the community.

Although incorporating multimodal digital features improved predictive performance relative to the baseline derived from demographic and questionnaire variables alone, the improvement was modest (approximately 5–8% in AUC and F1-score). This could only indicate that facial, acoustic, and linguistic signals provide incremental rather than transformative predictive value. The best performance came not from multimodal data alone but from its integration with conventional variables, suggesting these features should be understood as an additive to questionnaire-based assessment.

While most studies explored SI within the general population [38], the current study examined SI in MDD, given that depression is a major confounding factor in SI research. Although recent studies have reported strong performance of LLM-based approaches for suicide risk detection [39], LIWC remains preferable in this study due to its psychologically grounded and interpretable linguistic categories, which are suitable for biomarker identification. Some linguistic features, such as the category “reward” were identified to show strong correlations with SI in the study. It is postulated that people experiencing SI may focus on immediate relief from stress and depression as a coping mechanism, which may be reflected by their use of hedonistic-related (“reward”) and impulse-control-related (“reward”) category words such as pleasure, urge, temptation [40]. The study also found that individuals with MDD and SI used fewer first-person singular pronouns than those with MDD without SI. This may be because people with SI tend to focus on themselves in a more passive and detached manner. Instead of actively referencing themselves as the subject, they often cast themselves as the object in sentences, which reflects feelings of being acted upon and diminished sense of agency [41]. As a result, their language contains less explicit self-reference.

Furthermore, differences in paralinguistic features such as voice, pitch, rhythm and loudness have been proven to be significant biomarkers for detecting suicidality [42,43]. For example, the fundamental acoustic frequency in the SI group is lower than that of the non-suicidal group, which aligns with previous research indicating that a more monotonous voice reflects reduced energy and a depressed mood [44]. Feature importance of acoustic features such as Shimmer contributed to SI prediction in the model is also consistent with prior literature showing that acoustic biomarkers carry information relevant to psychiatric states, particularly depression-related alterations [45].

The importance of facial features such as AU12 and AU26 in our models is broadly consistent with prior work showing that depression-related signal is partly expressed through altered facial affect and reduced expressivity. Jiang et al. [46] found that happiness-related facial features were the most informative predictors in remission/non-response classification, with average and maximum happiness ranking highest and AU4 contributing negatively, supporting the relevance of affect-linked facial behavior in psychiatric assessment. Although the exact AUs differed, our findings similarly suggest that lower-face expressive actions may carry clinically meaningful information. Our multimodal findings are also aligned with Jiang et al. [47], who reported that facial, vocal, linguistic, and cardiovascular features extracted from remote interviews all contributed to mental health classification, with multimodal fusion outperforming most unimodal models. In particular, their findings of reduced facial expressivity, increased sadness in language, and relevant acoustic variation support the biological plausibility of our facial, linguistic, and acoustic predictors.

Our findings indicate a strong correlation between being divorced, never married, or widowed and the presence of SI. These results are consistent with most epidemiological and clinical studies that link marital status to suicidality [48]. It has long been established that subjective well-being and life satisfaction are closely related to suicide, a relationship that may be elucidated by socio-economic and ideological factors as previously discussed [49].

Several limitations of this study should be acknowledged. First, suicidality spectrum is complicated and is more than just a binary classification problem, this may have limited the model’s ability to accurately identify and detect these outcomes in real-world settings. Second, the baseline HDRS interview evaluates SI during the week immediately prior to EMA data collection and thus captures recent and relatively stable SI status. However, this approach does not allow us to investigate short-term fluctuations or predict next-day SI based on self-report measures [7]. Therefore, while our method provides a practical foundation for examining associations between digital features and recent SI in clinical populations [50], future research should strive for closer alignment of assessment timing to enhance the accuracy of such associations. Third, the sample size for both SI and non-SI MDD subjects were limited and with the absence of fully nested cross-validation, this could potentially constrain the robustness of model training and reducing the reliability of the validation results. Fourth, SI was operated as a binary outcome, which may have obscured clinically differences in severity. This choice was primarily driven by the imbalanced distribution of H11 severity levels in our sample, including relatively few higher-severity cases. Moreover, the high dimensionality of the feature space relative to the sample size still poses a risk of overfitting; although feature selection was conducted within the cross-validation framework to mitigate this, residual overfitting cannot be fully excluded. Finally, SI was assessed solely through clinical interviews rather than self-report measures, so this study cannot determine the clinical significance of differences between multimodal EMA and simply asking patients about suicidal thoughts.

To move toward meaningful clinical integration, future work should focus on larger, longitudinal datasets, inclusion of real-time behavioral or digital biomarkers, multiclass classification and careful attention to model fairness and ethical implications. Post-EMA assessment of SI should also be conducted to examine how well EMA-derived features correspond to suicidality assessed after the monitoring period, and how well it operates under fluctuation of suicidality.

Most importantly, the absence of external validation in independent cohorts remains the principal limitation of this study and fundamentally restricts the generalizability of the findings. As the models were developed within a single, relatively small cohort, their performance in other populations, settings, or languages cannot be ensured. The results should therefore be regarded as exploratory and hypothesis-generating rather than as evidence of generalizable clinical performance. Validation in larger, independent cohorts is essential before any conclusions regarding clinical utility or real-world deployment can be drawn. Although the findings demonstrate that machine learning approaches can achieve satisfactory performance in identifying patients with MDD with SI, several challenges remain regarding their implementation in real-world clinical settings. Suicide risk detection is complex, and even under controlled conditions, models typically achieve AUC values of approximately 0.80 during internal validation. In clinical practices, performance may be reduced, potentially resulting in missed cases or unnecessary interventions, both of which carry significant clinical risks. Nonetheless, such models may hold value as tools for community-level screening, or as decision-support tools rather than replacements for clinician judgment. Offering potential utility in preliminary risk identification and population-level monitoring. Nevertheless, models with moderate detection ability can still provide meaningful benefits when integrated into broader screening protocols, particularly in contexts where clinical resources are limited and early triage is essential. AI-assisted systems may support prioritization of high-risk patients for psychiatric evaluation and highlight subtle signals that warrant clinical attention. Their effective implementation, however, requires ongoing validation across diverse populations, improved interpretability of model outputs, and the establishment of clear clinical thresholds to guide action.

## Conclusion

Multimodal features are seen to have incremental value in identifying individuals with SI among MDD patients. Best models have achieved AUC between 0.70 and 0.80, demonstrating potential utility in identification. Additionally, this study is exploratory and serves as a proof-of-concept of use of multimodal features in suicide detection and identifies key multimodal features that may be relevant to the detection of suicidality. Further validation and investigation is needed before clinical utilities could be ensured.

## Supporting information

S1 Appendix Details of hyperparameters and feature selection results. Hyperparameter search combinations, best-performing parameter combinations for each model and results for various number of features selected.
(DOCX)

S2 Appendix Completed TRIPOD+AI guideline checklist. Completed TRIPOD+AI reporting guideline checklist for the transparent reporting of models. The TRIPOD+AI checklist was adapted from Collins GS, Moons KGM, Dhiman P, et al. TRIPOD+AI statement: updated guidance for reporting clinical prediction models that use regression or machine learning methods. BMJ (Clinical research ed). 2024;385:e078378. https://doi.org/10.1136/bmj-2023-078378, under the terms of the Creative Commons Attribution 4.0 International License (CC BY 4.0, https://creativecommons.org/licenses/by/4.0/). Changes were made to the original material.
(PDF)
