# Supplementary material for: Identification of suicidal ideation in major depressive disorder: A machine learning approach with multimodal digital features
Source: PLOS Digit Health. 2026 Aug 27;5(8):e0001615. doi: 10.1371/journal.pdig.0001615 (PMC13521398; doi:10.1371/journal.pdig.0001615)
Supplement: Supplementary file 2 [file pdig.0001615.s001.docx]

_Abbreviations:_ AUC = Area under the receiver operating characteristic curve; ANN = Artificial Neural Network, DT = Decision Tree, GB= Gradient Boost, KNN = k-Nearest Neighbors; LR = Logistic Regression, NB = Naive Bayes, RF = Random Forest, SVM = Support Vector Machine, CatBoost = Categorical Boosting, XG = Extreme Gradient Boosting

Table 1. Number of Features against Area Under Curve (AUC)

| No. features\AUC | ANN | Catboost | | DT | GB | KNN | LR | NB | RF | SVM | | XG |
| --- | --- | --- | --- | --- | --- | --- | --- | --- | --- | --- | --- | --- |
| 10 | 0.659 | 0.717 | 0.593 | | 0.532 | 0.711 | 0.695 | 0.612 | 0.676 | | 0.420 | 0.534 |
| 20 | 0.728 | 0.794 | 0.687 | | 0.612 | 0.754 | 0.634 | 0.720 | 0.651 | | 0.627 | 0.556 |
| 30 | 0.573 | 0.790 | 0.635 | | 0.586 | 0.748 | 0.669 | 0.664 | 0.639 | | 0.670 | 0.590 |
| 40 | 0.593 | 0.774 | 0.612 | | 0.593 | 0.747 | 0.646 | 0.660 | 0.612 | | 0.642 | 0.581 |
| 50 | 0.607 | 0.779 | 0.681 | | 0.501 | 0.788 | 0.646 | 0.635 | 0.605 | | 0.658 | 0.554 |
| 100 | 0.669 | 0.777 | 0.659 | | 0.551 | 0.781 | 0.635 | 0.608 | 0.613 | | 0.667 | 0.594 |
| 500 | 0.616 | 0.645 | 0.644 | | 0.579 | 0.611 | 0.639 | 0.587 | 0.640 | | 0.599 | 0.554 |
| 1009 | 0.489 | 0.624 | 0.554 | | 0.435 | 0.411 | 0.642 | 0.582 | 0.516 | | 0.590 | 0.565 |

Table 2. Number of Features against F1-score

| No. features\F1 | ANN | Catboost | DT | GB | KNN | LR | NB | RF | SVM | | XG |
| --- | --- | --- | --- | --- | --- | --- | --- | --- | --- | --- | --- |
| 10 | 0.542 | 0.667 | 0.487 | 0.463 | 0.632 | 0.631 | 0.536 | 0.559 | | 0.555 | 0.447 |
| 20 | 0.635 | 0.734 | 0.615 | 0.485 | 0.625 | 0.581 | 0.575 | 0.546 | | 0.506 | 0.481 |
| 30 | 0.432 | 0.699 | 0.525 | 0.427 | 0.642 | 0.630 | 0.600 | 0.547 | | 0.584 | 0.485 |
| 40 | 0.532 | 0.691 | 0.519 | 0.425 | 0.644 | 0.608 | 0.591 | 0.533 | | 0.571 | 0.507 |
| 50 | 0.586 | 0.677 | 0.598 | 0.378 | 0.709 | 0.596 | 0.523 | 0.435 | | 0.571 | 0.524 |
| 100 | 0.568 | 0.667 | 0.535 | 0.394 | 0.667 | 0.583 | 0.394 | 0.546 | | 0.548 | 0.514 |
| 500 | 0.544 | 0.576 | 0.537 | 0.419 | 0.551 | 0.583 | 0.456 | 0.555 | | 0.488 | 0.431 |
| 1009 | 0.509 | 0.557 | 0.420 | 0.350 | 0.492 | 0.578 | 0.523 | 0.485 | | 0.548 | 0.457 |

Table 3. Number of Features against Accuracy

| No. features\Accuracy | ANN | Catboost | | | DT | GB | KNN | LR | NB | RF | | SVM | | XG |
| --- | --- | --- | --- | --- | --- | --- | --- | --- | --- | --- | --- | --- | --- | --- |
| 10 | 0.556 | | 0.667 | 0.487 | | 0.463 | 0.647 | 0.586 | 0.546 | 0.505 | 0.384 | | 0.576 | |
| 20 | 0.687 | | 0.734 | 0.615 | | 0.485 | 0.636 | 0.606 | 0.657 | 0.596 | 0.606 | | 0.586 | |
| 30 | 0.576 | | 0.699 | 0.525 | | 0.427 | 0.606 | 0.596 | 0.596 | 0.515 | 0.626 | | 0.657 | |
| 40 | 0.626 | | 0.691 | 0.519 | | 0.425 | 0.687 | 0.596 | 0.566 | 0.647 | 0.606 | | 0.647 | |
| 50 | 0.515 | | 0.677 | 0.598 | | 0.378 | 0.768 | 0.576 | 0.576 | 0.475 | 0.606 | | 0.596 | |
| 100 | 0.586 | | 0.667 | 0.535 | | 0.394 | 0.647 | 0.465 | 0.596 | 0.495 | 0.616 | | 0.657 | |
| 500 | 0.576 | | 0.576 | 0.537 | | 0.419 | 0.505 | 0.465 | 0.566 | 0.384 | 0.556 | | 0.626 | |
| 1009 | 0.414 | | 0.557 | 0.420 | | 0.350 | 0.354 | 0.455 | 0.576 | 0.485 | 0.434 | | 0.616 | |

Table 4. Number of Features against sensitivity

| No. features\ sensitivity | ANN | Catboost | DT | GB | KNN | LR | NB | RF | | SVM | | XG |
| --- | --- | --- | --- | --- | --- | --- | --- | --- | --- | --- | --- | --- |
| 10 | 0.684 | 0.790 | 0.500 | 0.500 | 0.790 | 0.921 | 0.684 | 0.816 | 1.000 | | 0.447 | |
| 20 | 0.711 | 0.763 | 0.842 | 0.421 | 0.790 | 0.711 | 0.605 | 0.632 | 0.526 | | 0.500 | |
| 30 | 0.421 | 0.763 | 0.553 | 0.421 | 0.921 | 0.895 | 0.790 | 0.763 | 0.684 | | 0.421 | |
| 40 | 0.553 | 0.763 | 0.553 | 0.447 | 0.737 | 0.816 | 0.816 | 0.526 | 0.684 | | 0.474 | |
| 50 | 0.895 | 0.553 | 0.684 | 0.368 | 0.737 | 0.816 | 0.605 | 0.526 | 0.684 | | 0.579 | |
| 100 | 0.711 | 0.921 | 0.500 | 0.368 | 0.921 | 0.974 | 0.342 | 0.790 | 0.605 | | 0.474 | |
| 500 | 0.658 | 0.895 | 0.579 | 0.342 | 0.790 | 0.974 | 0.474 | 1.000 | 0.553 | | 0.368 | |
| 1009 | 0.790 | 0.579 | 0.447 | 0.368 | 0.816 | 0.974 | 0.605 | 0.632 | 0.895 | | 0.421 | |

Table 5. Number of Features against Specificity

| No. features\ Specificity | ANN | Catboost | DT | GB | KNN | LR | NB | RF | | SVM | | XG |
| --- | --- | --- | --- | --- | --- | --- | --- | --- | --- | --- | --- | --- |
| 10 | 0.475 | 0.639 | 0.656 | 0.590 | 0.557 | 0.377 | 0.459 | 0.312 | 0.000 | | 0.656 | |
| 20 | 0.672 | 0.803 | 0.443 | 0.803 | 0.541 | 0.541 | 0.689 | 0.574 | 0.656 | | 0.639 | |
| 30 | 0.672 | 0.738 | 0.656 | 0.656 | 0.410 | 0.410 | 0.475 | 0.361 | 0.590 | | 0.803 | |
| 40 | 0.672 | 0.721 | 0.639 | 0.590 | 0.656 | 0.459 | 0.410 | 0.721 | 0.557 | | 0.754 | |
| 50 | 0.279 | 0.951 | 0.623 | 0.639 | 0.787 | 0.426 | 0.557 | 0.443 | 0.557 | | 0.607 | |
| 100 | 0.508 | 0.475 | 0.771 | 0.689 | 0.475 | 0.148 | 0.754 | 0.312 | 0.623 | | 0.771 | |
| 500 | 0.525 | 0.246 | 0.639 | 0.820 | 0.328 | 0.148 | 0.623 | 0.000 | 0.557 | | 0.787 | |
| 1009 | 0.180 | 0.689 | 0.574 | 0.541 | 0.066 | 0.131 | 0.557 | 0.393 | 0.148 | | 0.738 | |

Table 6. Number of Features against NPV

| No. features\ NPV | ANN | Catboost | DT | GB | KNN | LR | NB | RF | | SVM | | XG |
| --- | --- | --- | --- | --- | --- | --- | --- | --- | --- | --- | --- | --- |
| 10 | 0.707 | 0.830 | 0.678 | 0.655 | 0.810 | 0.885 | 0.700 | 0.731 | 0.000 | | 0.656 | |
| 20 | 0.789 | 0.845 | 0.818 | 0.690 | 0.805 | 0.750 | 0.737 | 0.714 | 0.690 | | 0.672 | |
| 30 | 0.651 | 0.833 | 0.702 | 0.645 | 0.893 | 0.862 | 0.784 | 0.710 | 0.750 | | 0.690 | |
| 40 | 0.707 | 0.830 | 0.696 | 0.632 | 0.800 | 0.800 | 0.781 | 0.710 | 0.739 | | 0.697 | |
| 50 | 0.810 | 0.773 | 0.760 | 0.619 | 0.828 | 0.788 | 0.694 | 0.600 | 0.739 | | 0.698 | |
| 100 | 0.738 | 0.906 | 0.712 | 0.636 | 0.906 | 0.900 | 0.639 | 0.704 | 0.717 | | 0.702 | |
| 500 | 0.711 | 0.790 | 0.709 | 0.667 | 0.714 | 0.900 | 0.655 | 0.000 | 0.667 | | 0.667 | |
| 1009 | 0.579 | 0.724 | 0.625 | 0.579 | 0.364 | 0.889 | 0.694 | 0.632 | 0.692 | | 0.672 | |

Table 7. Number of Features against PPV

| No. features\ PPV | ANN | Catboost | DT | GB | KNN | LR | NB | RF | | SVM | | XG |
| --- | --- | --- | --- | --- | --- | --- | --- | --- | --- | --- | --- | --- |
| 10 | 0.448 | 0.577 | 0.475 | 0.432 | 0.526 | 0.480 | 0.441 | 0.425 | 0.384 | | 0.447 | |
| 20 | 0.575 | 0.707 | 0.485 | 0.571 | 0.517 | 0.491 | 0.548 | 0.480 | 0.488 | | 0.463 | |
| 30 | 0.444 | 0.644 | 0.500 | 0.432 | 0.493 | 0.486 | 0.484 | 0.427 | 0.510 | | 0.571 | |
| 40 | 0.512 | 0.630 | 0.488 | 0.405 | 0.571 | 0.484 | 0.463 | 0.541 | 0.491 | | 0.546 | |
| 50 | 0.436 | 0.875 | 0.531 | 0.389 | 0.683 | 0.470 | 0.460 | 0.370 | 0.491 | | 0.478 | |
| 100 | 0.474 | 0.522 | 0.576 | 0.424 | 0.522 | 0.416 | 0.464 | 0.417 | 0.500 | | 0.563 | |
| 500 | 0.463 | 0.425 | 0.500 | 0.542 | 0.423 | 0.416 | 0.439 | 0.384 | 0.438 | | 0.519 | |
| 1009 | 0.375 | 0.537 | 0.395 | 0.333 | 0.352 | 0.411 | 0.460 | 0.393 | 0.395 | | 0.500 | |

Table 8. best hyperparameter from grid search

| Models | Best Parameters |
| --- | --- |
| ANN | activation: tanh  alpha: 0.0001  hidden_layer_sizes: (100, 50)  learning_rate: constant  max_iter: 500  solver: adam |
| DT | ccp_alpha: 0.001  class_weight: balanced  criterion: gini  max_depth: None  max_features: sqrt  min_samples_leaf: 2  min_samples_split: 10  splitter: random |
| GB | learning_rate: 0.1  max_depth: 7  max_features: sqrt  min_samples_leaf: 1  min_samples_split: 10  n_estimators: 100 |
| KNN | algorithm: auto  leaf_size: 10  metric: manhattan  n_neighbors: 5  p: 1  weights: uniform |
| Models | **Best Parameters** |
| LR | classifier__C: 0.01  classifier__class_weight: balanced  classifier__l1_ratio: 0.1  classifier__max_iter: 1000  classifier__penalty: l2  classifier__solver: liblinear |
| NB | alpha: 0.1  binarize: 0.0  fit_prior: True  n_features_to_select: 20  nb_type: gaussian  use_feature_selection: True  var_smoothing: 1e-09 |
| RF | classifier__bootstrap: True  classifier__class_weight: balanced  classifier__criterion: gini  classifier__max_depth: None  classifier__max_features: sqrt  classifier__min_samples_leaf: 1  classifier__min_samples_split: 10  classifier__n_estimators: 100 |
| SVM | C: 0.5  class_weight: balanced  degree: 2  gamma: scale  kernel: rbf |
| Catboost | auto_class_weights: None  depth: 2  iterations: 200  l2_leaf_reg: 7  learning_rate: 0.1  min_data_in_leaf: 1 |
| XG | n_estimators: 100  learning_rate: 0.3  max_depth: 3  min_child_weight: 1  gamma: 0  reg_alpha: 0.5  reg_lambda: 2  scale_pos_weight: 2 |

Table 9. Grid Search

| Models | Parameters |
| --- | --- |
| ANN | ann_param_grid = { 'hidden_layer_sizes': [(50,), (100,), (50, 50), (100, 50), (100, 100)], 'activation': ['relu', 'tanh', 'logistic'], 'solver': ['adam', 'lbfgs', 'sgd'], 'alpha': [0.0001, 0.001, 0.01, 0.1], 'learning_rate': ['constant', 'adaptive'], 'max_iter': [500, 1000] } |
| DT | dt_param_grid = { 'max_depth': [None, 10, 20, 30], 'min_samples_leaf': [1, 2, 4, 8], 'min_samples_split': [2, 5, 10], 'max_features': ['sqrt', 'log2'], 'criterion': ['gini', 'entropy'], 'splitter': ['best', 'random'], 'class_weight': [None, 'balanced','balanced_subsample'], 'ccp_alpha': [0.001, 0.01, 0.05] } |
| GB | gb_param_grid = { 'n_estimators': [100, 200, 300, 500], 'learning_rate': [0.01, 0.1, 0.2, 0,3, 1], 'max_depth': [3, 5, 7, 10], 'min_samples_split': [2, 5, 10], 'min_samples_leaf': [1, 2, 4, 8], 'max_features': ['sqrt', 'log2', None] } |
| KNN | svm_param_grid = { 'C': [0.01, 0.1, 1, 10, 100, 50, 15, 0.5, 5], 'kernel': ['linear', 'rbf', 'poly'], 'gamma': ['scale', 'auto', 0.001, 0.01, 0.1, 1, 0.5, 0.0001], 'degree': [2, 3, 4, 6, 8], # Only for poly kernel 'class_weight': [None, 'balanced','balanced_subsample'] } |
| LR | logistic_param_grid = { 'C': [0.001, 0.01, 0.1, 1, 10, 100], 'penalty': ['l1', 'l2', 'elasticnet'], 'solver': ['liblinear', 'saga', 'lbfgs'], 'l1_ratio': [0.1, 0.5, 0.7, 0.9], # Only for elasticnet 'class_weight': [None, 'balanced'], 'max_iter': [1000, 2000, 5000] } |
| NB | nb_param_grid = { 'nb_type': ['gaussian', 'multinomial', 'bernoulli'], 'var_smoothing': [1e-9, 1e-8, 1e-7, 1e-6, 1e-5], 'alpha': [0.1, 0.5, 1.0, 2.0], 'fit_prior': [True, False], 'binarize': [0.0, 0.5, None] } |
| RF | rf_param_grid = { 'n_estimators': [100, 200, 300], 'max_depth': [None, 10, 20, 30], 'max_features': ['sqrt', 'log2'], 'min_samples_leaf': [1, 2, 4, 8], 'min_samples_split': [2, 5, 10], 'bootstrap': [True, False], 'class_weight': ['balanced', 'balanced_subsample', None], 'criterion': ['gini', 'entropy'] } |
| SVM | svm_param_grid = { 'C': [0.01, 0.1, 1, 10, 100, 50, 15, 0.5, 5], 'kernel': ['linear', 'rbf', 'poly'], 'gamma': ['scale', 'auto', 0.001, 0.01, 0.1, 1, 0.5, 0.0001], 'degree': [2, 3, 4, 6, 8], # Only for poly kernel 'class_weight': [None, 'balanced','balanced_subsample'] } |
| Catboost | catboost_param_grid = { 'iterations': [200, 500, 800], 'learning_rate': [0.03, 0.1], 'depth': [2, 5], 'l2_leaf_reg': [3, 7], 'min_data_in_leaf': [1, 3, 5], 'auto_class_weights': [None, 'Balanced'] } |
| XG | xgboost_param_grid = { 'n_estimators': [100, 200, 300], 'learning_rate': [0.3, 0.1, 0.2], 'max_depth': [3, 5, 8], 'min_child_weight': [1, 3, 5], 'gamma': [0, 0.1, 0.2], 'reg_alpha': [0, 0.5], 'reg_lambda': [1, 2], 'scale_pos_weight': [2, 5, 10] } |
