# Supplementary material for: Identification of suicidal ideation in major depressive disorder: A machine learning approach with multimodal digital features
Source: PLOS Digit Health. 2026 Aug 27;5(8):e0001615. doi: 10.1371/journal.pdig.0001615 (PMC13521398; doi:10.1371/journal.pdig.0001615)
Supplement: Supplementary file 3 [file pdig.0001615.s004.docx]

Dear Editor and Reviewers,

We would like to express our gratitude for your comments and suggestions. These have allowed us to substantially improve our paper. We have carefully looked at all the issues raised in the reviews, and we hope we have sufficiently addressed them in this revision. In this document, we discuss our responses to the comments in detail. Thank you for your precious time and insightful suggestions.

Yours sincerely,

Authors

**Comments from Reviewer 1**

**Comment R1.1**

I wish to thank the authors for their valuable contribution to the important field of suicide prevention that continues to elude researchers and health officials. I further wish to highlight the quality and expertise found in the submitted work. The multi-modal assessment is a clear strong point that brings together many known risk factors for suicidal ideation, and I encourage the collaborators to distribute the underlying software widely.

In the following I provide comments that are mainly aimed at clarifying some aspects of the manuscript.

Major Comments

1) The larger question that will decide if such digital modality EMA are worth using in research and practice is if we can predict suicidal ideation more accurately than if we simply ask patients to self-report their current suicidal ideation. As I understand it, suicidal ideation was not assessed during the EMAs and this comparison not the goal of this study. It would be important to discuss the reasoning for these choices in the paper more clearly. This can maybe also be addressed by outlining more clearly how the authors view the role of this technology in patient care of the future.

**Response**

We thank the reviewer for pointing out this issue. The study aims to use EMA to gather multimodal data from patients in their daily lives for detecting clinician-rated suicidal ideation. In this study, we used clinical interviews as the outcome measure rather than self-report items/scales, as clinical interviews are more in-depth and are generally considered the gold standard for assessment. Compared with clinical interviews, self-report is a more direct way to assess suicidal ideation, but the reporting may at times be incomplete and influenced by reporting bias, including underreporting or avoidance. Still, there was no direct comparison of the predictive utility of multimodal EMA with simply asking patients about suicidal thoughts, which warrants further investigation in future research. This limitation has been acknowledged and added in this revised manuscript. The added texts are as follows:

“Finally, SI was assessed solely through clinical interviews rather than self-report measures, so this study cannot determine the clinical significance of differences between multimodal EMA and simply asking patients about suicidal thoughts.” (page 25)

**Comment R1.2**

2) The Introduction is quite short and could benefit from discussing the cited literature in greater detail, to place it more clearly in the greater research context. Also the question of ‘why are we looking at suicidal ideation instead of suicidal behavior’ is currently not addressed.

**Response**

We consider suicidal ideation, as a significant risk factor for suicidality, to be an appropriate outcome measure because it typically emerges before suicidal behavior, enabling earlier identification of risk. We have also provided a more comprehensive discussion of the cited literature. The added texts are as follows:

“Evidence from studies in other mental disorders further suggests that these multimodal approaches may be valuable for detection and clinical risk prediction.[10, 11] Despite these advancements, multimodal analysis for suicidal ideation (SI) detection remains limited. A recent review found that only 3 out of 14 multimodal, speech-based studies focused specifically on SI detection.[12] While suicidal ideation typically occurs before suicidal behaviors, these methods have the potential to identify risk at an earlier stage, enhancing suicide prevention efforts.

Recent machine learning studies have demonstrated the potential of diverse data modalities for suicide-related risk stratification in MDD and other psychiatric populations[12]. Whole-brain functional connectivity has been used to distinguish patients with high versus low suicide risk[13], while dynamic functional connectivity and connectomics-based resting-state network analyses have further suggested that suicidality is associated with disruption of functional brain organization.[14, 15] Other studies have shown that integrating pain measures with event-related potential features[16] or combining neuroimaging with clinical text[17] may improve the classification of suicide attempt or suicidal ideation in MDD. In addition, hospital-based models using demographic, clinical, and biological variables have identified correlates of suicidal ideation in MDD[18], and natural language processing of unstructured clinical notes has provided incremental predictive value beyond electronic health record variables in suicide prediction models.[19] However, many existing models rely on resource-intensive or difficult-to-scale data sources, and relatively little is known about whether real-life, multimodal EMA-derived features could improve the identification of suicidal ideation in individuals with MDD.” (Page 5)

**Comment R1.3**

3) The data availability statement is currently insufficient. Please discuss in more detail what data could be potentially be shared and how. While the recorded videos naturally cannot be shared, maybe facial action units or aggregate measures thereof be shared? Some clinical information and timestamps may need to be perturbed or fully removed to ensure anonymity.

**Response**

We thank the reviewer for the suggestion. The dataset supporting this study is not currently available due to the ongoing nature of the project. We will share the full data upon reasonable request, in accordance with institutional and journal policies. The revised data availability statement is as follows:

“Data can be requested to the following: The institutional contact: Li Chiu Kong Family Sleep Assessment Unit sleepresearch@cuhk.edu.hk

Full citation of the data: Li Chiu Kong Family Sleep Assessment Unit. DMoMo data. The Chinese University of Hong Kong: Hong Kong, China; 2025 [cited 2025 Dec 18].

Available from: sleepresearch@cuhk.edu.hk.

The third-party organization - ethics committee: You can email the Joint CUHK-NTEC Clinical Research Ethics Committee at crec@cuhk.edu.hk for inquiries about our clinical research ethics.” (Page 28)

**Comment R1.4**

4) 350 – 353: There is some grammatical confusion here, which should be clarified. The word “me” is a first-person pronoun just as much as “I” and therefore “I am sad” and “They upset me” both contain a first-person pronoun. It is important to confirm exactly how this was coded in LWIC. If “They upset me” does not contain a first-person pronoun in Cantonese, then please describe this.

**Response**

In Cantonese, the first-person pronouns “I” and “me” are not morphologically distinguished and are typically represented by the same character “我” (or equivalent variants). In the Chinese LIWC-style dictionaries, first-person singular forms such as “我” are coded in the first-person singular pronoun category[1]. Therefore, expressions such as “I am sad” and “They upset me” both contain a first-person pronoun in Cantonese and were consistently coded as such. To avoid confusion, we have revised the text as follows:

“The study also found that individuals with MDD and SI used fewer first-person singular pronouns than those with MDD without SI. This may be because people with SI tend to focus on themselves in a more passive and detached manner. Instead of actively referencing themselves as the subject, they often cast themselves as the object in sentences, which reflects feelings of being acted upon and diminished sense of agency.[41] As a result, their language contains less explicit self-reference.” (Page 23)

Ref[1]: Huang CL, Chung CK, Hui N, Lin YC, Seih YT, Lam BCP, et al. The development of the Chinese linguistic inquiry and word count dictionary. Chinese Journal of Psychology. 2012;54(2):185–201.

**Comment R1.4**

5) Missing data is reported to be 0.05% (line 197), but it is unclear what that means. Does this mean that we have 99.95% rate of responding to EMA prompts? 99 participants x 7 days x 4 EMAs per day = 2772 responses. 0.05% missing would mean only one response (rounded) is missing. Please clarify if this is accurate in the text as it would constitute a phenomenal compliance rate. As feasibility of such technology is an important insight produced by this study, this should also be brought up in the discussion.

**Response**

We agree that demonstrating feasibility is an important contribution of this study. The reported 0.05% missing data does not include any EMA data; rather, the missing data were limited to demographic and questionnaire items, such as income level (which some participants may have chosen not to disclose) or individual questionnaire items that were skipped. There was no missing data in the aggregated EMA datasets used for analysis. To clarify, the added texts are as follow:

“Missing data (0.05%), limited to demographic and questionnaire information, were addressed using K-Nearest Neighbours (KNN) imputation.” (Page 9)

**Comment R1.5**

6) This paper does not require p-values. Table 1 should never contain p-values, as they do not aid interpretation and do not test a hypothesis; for reference see Cole et al. (2025)Ref. This extends in this paper to Table 2 as well. Further, I would also advise against reporting the p-value of the AUC. While not mathematically incorrect, it does not aid interpretation and is certainly unusual. In Tables 3 & 4 it could be argued that p-values make sense as the differences here are less clearly established, however in that case the p-values need to be corrected for multiple comparisons. Alternatively, it could be explained why there was not correction for multiple comparisons.

Ref.: Cole, J. B., Knack, S. K. S., & Driver, B. E. (2025). The value of P-values in "Table 1". The American journal of emergency medicine, 100, 182–186. Advance online publication. https://doi.org/10.1016/j.ajem.2025.12.003

**Response**

For Table 1,2 and 5, we are grateful for the comment and have removed the p-values.

Regarding Tables 3 and 4, we appreciate the reviewer’s point about multiple comparisons. The primary reason we did not apply a multiple testing correction (such as Bonferroni) is due to the high-dimensional nature of our feature space, which includes thousands of variables. In this supervised machine learning context, standard family-wise error rate corrections become overly conservative. Applying them would severely inflate Type II errors (false negatives), effectively pushing all adjusted p-values to 1.0 and obscuring any potentially relevant features.

The analyses in Tables 3 and 4 were exploratory and aimed to identify candidate features within a high-dimensional feature space, p-values are reported for descriptive screening purposes and should be interpreted as an exploratory rather than confirmatory. The following text are added to avoid confusion:

“P-values are presented for exploratory and descriptive purposes rather than strict hypothesis testing.” (Page 11)

**Comment R1.6**

7) The feature selection aspect feels underdeveloped. A few more words on the reasoning why it was done or if there were patterns in the optimal amount of features would be welcome. Further, it would be of high interest to learn which features were the strongest predictors. This could very valuable to follow-up research.

**Response**

The rationale for including feature selection was to address the high dimensionality of the dataset relative to the sample size, reducing noise, improve model interpretability and stability. Machine-learning approaches are well suited to high-dimensional datasets, and feature selection is commonly used in this setting to reduce noise and redundancy, improve model performance, and identify a smaller subset of informative predictors. [2] Across all models, the optimal number of selected features were in the range of 10–50, suggesting that a relatively small subset of variables contained most of the predictive signal. The details are listed in the appendix.

To further investigate, the feature importance was discussed in the revised manuscript. The following text are added:

“Based on the feature importance of the best performing models, the predictive information was distributed across multiple modalities. In the CatBoost model, the strongest predictors included facial, acoustic, linguistic variables, and several HADS items, with AU26, Shimmer, and AU12 ranking highest.

In the Naive Bayes model, questionnaire variables were relatively more prominent, but multimodal variables also showed relevant importance, including linguistic variable “informal”, Shimmer and AU26.

Facial features such as AU12 and AU26, linguistic features including negative emotion, time, and informality, as well as the acoustic variable shimmer, consistently showed relative importance across the models. Greater contribution by facial and linguistic features are seen over acoustic features. Overall, the findings support a multimodal contribution to prediction, with the noticeable multimodal features.” (Page 18-19)

Ref[2]: P. R. Anukrishna, V. Paul. A review on feature selection for high dimensional data. International Conference on Inventive Systems and Control (ICISC). 2017; pp. 1-4. doi: 10.1109/ICISC.2017.8068746.

**Comments from Reviewer 2**

**Comment R2.1**

1. Data Inconsistency and Integrity. There is a numerical inconsistency between the Abstract and the Results section that undermines confidence in the reported statistics. The abstract: Reports 31 patients with SI and 61 without SI (Total N = 92). The Results (and Table 1): Reports 38 patients with SI and 61 without SI (Total N = 99). It is unclear which dataset was used for the training and testing. If the abstract numbers are from a previous iteration, the abstract was not updated. If the results numbers are correct, the abstract is factually wrong. This must be resolved.

**Response**

We sincerely thank the reviewer for this pointing this out. The correct total sample size is N = 99, comprising 38 participants with suicidal ideation (SI) and 61 without SI. The previously reported number in the Abstract (N = 92) was a typo and has now been corrected in the manuscript.

**Comment R2.2**

2. Overfitting risk. The study utilizes 99 subjects (at best) but extracts 1009 features. Even with the reported feature selection (ANOVA F-value), the ratio of observations to predictors is extremely low. While the authors use cross-validation (5-fold) and feature selection inside the fold (which is good practice to avoid leakage), the high dimensionality of the initial pool increases the "degrees of freedom" for finding spurious correlations. The effective sample size for the minority class (SI) is only ~38. In such small N studies, performance metrics like AUC often exhibit high variance. The reported AUC of 0.79 might not generalize to new data.

Also, investigating model performance using only features from survey and demographics to predict the H11 label (without the multi-modal data/features) would provide a baseline to compare and decide the contribution of multimodal data and to see how/if it improves the performance.

**Response**

We acknowledge the reviewer’s concern regarding the risk of overfitting given the relatively small sample size and high-dimensional feature space. For this purpose, we attempt using cross-validation to address overfitting. We also strengthened the limitations section regarding the small sample size and also added feature importance to increase the interpretability of the models. The following text are added:

“Based on the feature importance of the best performing models, the predictive information was distributed across multiple modalities. In the CatBoost model, the strongest predictors included facial, acoustic, linguistic variables, and several HADS items, with AU26, Shimmer, and AU12 ranking highest.

In the Naive Bayes model, questionnaire variables were relatively more prominent, but multimodal variables also showed relevant importance, including linguistic variable “informal”, Shimmer and AU26.

Facial features such as AU12 and AU26, linguistic features including negative emotion, time, and informality, as well as the acoustic variable shimmer, consistently showed relative importance across the models. Greater contribution by facial and linguistic features are seen over acoustic features. Overall, the findings support a multimodal contribution to prediction, with the noticeable multimodal features.” (Page 18-19)

“The reported results should be interpreted as exploratory rather than as evidence of generalizable clinical performance.” (page 25)

Apart from AUC, the study also reports other metrics including F1 score, accuracy, sensitivity, specificity, positive predictive value, and negative predictive value for a more comprehensive evaluation of the models.

In addition, we also added a baseline analysis using only survey and demographic variables to allow a more direct evaluation of the incremental contribution of multimodal features. The following text are added:

“To assess whether multimodal digital features added predictive value beyond conventional clinical information, we compared models trained on demographic and questionnaire variables (Table 6) alone with models trained on broader feature sets including multimodal data. The best performance was obtained using the combined demographic, questionnaire, and multimodal with CatBoost achieving an AUC of 0.79, F1 score of 0.73, and accuracy of 0.79. In comparison, the best baseline model using demographic and questionnaire features alone achieved lower performance. The difference between the baseline model and multimodal models is 5-8% in term of AUC and F1-score, greater improvement is seen across accuracy, specificity, PPV and NPV.This pattern indicates that multimodal features provide potential incrementative predictive information beyond standard self-report and demographic measures. Importantly, the highest performance was not obtained from multimodal data alone, but from their integration with conventional variables, suggesting that multimodal feature could be useful as an additive to questionnaire-based assessment.

An additional pattern was observed across model types. Baseline models using demographic and questionnaire features tended to perform relatively well with simpler models such as logistic regression and random forest, whereas models incorporating multimodal features usually achieved stronger performance with more flexible approaches such as ANN and CatBoost. Although this pattern should be interpreted cautiously, it may suggest that the relationship between multimodal features and SI is more complex and potentially more nonlinear than questionnaire and demographic features, requiring models that can better capture feature interactions and higher-order structure.” (page 19-21)

**Comment R2.3**

3. Label Definition and Temporal Mismatch. The target label (SI) is defined by a single item (H11) from the HDRS interview conducted at baseline. The features are collected via EMA over the following 7 days. Suicidal ideation is known to be transient and fluctuating. Labeling a full week of digital data based on a single "snapshot" interview conducted previously introduces significant label noise. A patient might have had SI during the interview but not during the app usage, or vice versa. The authors acknowledge this in limitations, but it fundamentally weakens the supervised learning setup. Moreover, it would be somewhat more reliable if this single item were collected after the 7 days of daily interactions/data collection, since it asks about thoughts from the last week.

**Response**

We acknowledge concerns about the temporal mismatch between the baseline SI assessment and the subsequent 7-day EMA data collection. However, using the baseline HDRS SI interview as our outcome measure remains appropriate, as it captures a more stable, clinically rated SI rather than the more variable self-reported SI. The digital features obtained are likely to reflect the recent SI status, which is measured via in-depth clinical interview and tends to be more stable. Importantly, our study does not aim to predict short-term fluctuations in SI, such as next-day SI based on self-reports. While we recognize this limitation and recommend closer alignment of assessment timing in future studies, our approach provides a practical and reasonable basis for examining associations between digital features and recent SI in clinical populations. The added texts are as follows:

“Second, the baseline HDRS interview evaluates SI during the week immediately prior to EMA data collection and thus captures recent and relatively stable SI status. However, this approach does not allow us to investigate short-term fluctuations or predict next-day SI based on self-report measures.[7] Therefore, while our method provides a practical foundation for examining associations between digital features and recent SI in clinical populations[49], future research should strive for closer alignment of assessment timing to enhance the accuracy of such associations.” (Page 24)

“Post-EMA assessment of suicidal ideation should also be conducted to examine how well EMA-derived features correspond to suicidality assessed after the monitoring period, and how well it operates under fluctuation of suicidality.” (Page 25)

**Comment R2.4**

4. Validation issues and missed opportunities on public data. The study relies entirely on internal 5-fold cross-validation. Given the small sample size and high feature count, internal CV is prone to optimistic bias. Without an independent hold-out set, the claim of "robust" performance is unsupported. The use of Mann-Whitney tests to compare AUCs across models on the same dataset (correlated samples) is often statistically underpowered or inappropriate compared to tests like DeLong’s test. The study relies exclusively on a small, private internal dataset (N=99?). The authors failed to leverage (through transfer learning) or benchmark against standard public datasets used in this domain.

Relevant Public Databases (Missing from Study):

DAIC-WOZ (Distress Analysis Interview Corpus - Wizard of Oz): This is the "gold standard" public dataset for multimodal depression detection, containing audio, video, and text of clinical interviews. The authors should have used this for transfer learning or as an external validation set.

EATD-Corpus (Emotional Audio-Textual Depression Corpus): A dataset specifically for depression detection using audio and text, highly relevant for their acoustic/linguistic analysis.

MODMA (Multi-modal Open Dataset for Mental-disorder Analysis): Includes EEG and audio from depressed patients, relevant for cross-modal comparisons.

**Response**

We thank the reviewer for suggesting public dataset for external validation, the absence of an independent external dataset is a limitation of this study and have certainly limited the generalizability of the models. Currently, the lack of external validation was mainly because the public datasets are not directly aligned with our study design. Specifically, our task focuses on suicidal ideation within patients with MDD, whereas these public datasets are largely designed for depression detection/severity or other related tasks. In addition, our cohort consists of Chinese-speaking participants, and both linguistic and acoustic features may vary substantially across languages. Finally, our data were collected using a smartphone-based EMA protocol with multimodal features in a naturalistic setting, which differs from the structured interview settings and available modalities of the datasets. For these reasons, we refrained from claiming model robustness to avoid overstating our findings.

The difference between the baseline model and multimodal models is 5-8% in term of AUC and F1-score, greater improvement is seen across accuracy, specificity, PPV and NPV. However, given the small sample size, the analyses were exploratory, p-values should be interpreted as an exploratory rather than confirmatory. Please refer to Comment R1.5. The following text are added to avoid confusion:

“P-values are presented for exploratory and descriptive purposes rather than strict hypothesis testing.” (Page 11)

**Comment R2.5**

B. Novelty issues and lack of appropriate citations and framing in terms of the existing literature.

1. The novelty of this work is low to moderate. The specific application of these multimodal features to Suicidal Ideation (SI) appears to be a new analysis. However, the data collection platform (D-MOMO), the study protocol, the participant pool (Hong Kong Chinese MDD patients), and the feature extraction pipeline (OpenFace, OpenSMILE, LIWC) have been previously published by this research group. This might be interpreted as salami-slicing of a dataset previously used to predict MDD diagnosis and remission status. The authors should explicitly state if this is a secondary analysis of the same cohort, and what is new in this approach.

**Response**

We appreciate the reviewer highlighting this issue, which allows us to provide further clarification. Our study makes an important clinical contribution by introducing a novel, feasible self-assessment tool for SI, validated against clinical assessments as the gold standard. Regarding how the work differs from previous ones, earlier multimodal studies primarily used clinician-/interviewer-led in-person or telehealth interview protocols (e.g., Zoom-based remote interviews)[3-6], whereas our study collected EMA, questionnaires, and video entirely through a patient-facing mobile app, in real-world everyday settings, and without requiring synchronous clinician/research personnel involvement during collection. To clarify, we have extended the discussion for this part in the revised manuscript:

“Importantly, these earlier multimodal approaches were generally based on hospital-derived data or clinician guided interview or different assessment protocols, whereas our study collected EMA responses and questionnaires entirely through a mobile application in real-world daily settings, without requiring involvement of synchronous clinician/research personnel during data acquisition, in a more accessible and naturalistic setting.”(Page 22)

Indeed, Chen et al. (2024) and the present manuscript arise from the same broader D-MOMO project, but they address different research questions. Chen et al. focused on depression/MDD-related assessment and did not investigate suicidality, whereas the present study specifically examines suicidal ideation/risk within depressed patients. In addition, this is not merely a re-analysis of the identical sample: the D-MOMO project is ongoing, and the current study includes an expanded MDD cohort (99 vs 61). We therefore revised the manuscript to explicitly state that the present work was part of the larger D-MOMO project and clarified the research question:

“This study examines the utility of a multimodal EMA-machine learning framework for the identification of clinically rated SI in individuals diagnosed with MDD, with the objective of contributing to more accurate risk stratification, assessing incremental value of multimodal features and facilitating timely intervention in real-world settings.” (Page 5-6)

“This research is an on-going study aimed at developing a digital mental health platform for diagnosing and assessing MDD subjects.[8, 9] The present report represents an interim analysis of the data collected between June 2021 and January 2025.” (Page 6)

Ref[3-6]:

3.	Jiang Z, Harati S, Crowell A, Mayberg HS, Nemati S, Clifford GD. Classifying Major Depressive Disorder and Response to Deep Brain Stimulation Over Time by Analyzing Facial Expressions. IEEE Trans Biomed Eng. 2021;68(2). doi: 10.1109/TBME.2020.3010472.

4.	Jiang Z, Seyedi S, Griner E, Abbasi A, Rad AB, Kwon H,et al. Multimodal Mental Health Digital Biomarker Analysis From Remote Interviews Using Facial, Vocal, Linguistic, and Cardiovascular Patterns. IEEE J Biomed Health Inform. 2024;28(3):1680-91. doi: 10.1109/JBHI.2024.3352075.

5.	Cotes RO, Boazak M, Griner E, et al. Multimodal Assessment of Schizophrenia and Depression Utilizing Video, Acoustic, Locomotor, Electroencephalographic, and Heart Rate Technology: Protocol for an Observational Study. JMIR Res Protoc. 2022;11(7):e36417. doi: 10.2196/36417.

6.	Qin R CR, Yang K, Abbasi A, Dobolyi D, Seyedi S, et al. Language Models for Online Depression Detection: A Review and Benchmark Analysis on Remote Interviews. ACM Transactions on Management Information Systems. 2025;16(2):1-35. doi: 10.1145/3673906.

**Comment R2.6**

2. Relevant Prior Publications are not cited. For instance, I'm very surprised that the authors missed the following:

The dataset and methods (D-MOMO, 7-day EMA, same features) appear identical to: Chen J, Chan NY, Li CT, Chan JWY, Liu Y, Li SX, et al. "Multimodal digital assessment of depression with actigraphy and app in Hong Kong Chinese." Translational Psychiatry. 2024;14(1):1-8. doi: 10.1038/s41398-024-02873-4. In the Chen et al. (2024) paper, the same group used the D-MOMO app on 61 MDD subjects and 47 controls to detect MDD. The current manuscript appears to slightly expand the recruitment (N=99) or combine groups to focus on the SI sub-question.

The following background articles and previous work predate this work and should be reviewed and cited:

The "Gold Standard" Dataset & Protocols (Missing Benchmarks)

The authors claim their multimodal video approach is novel, yet they fail to cite the SimSensei Kiosk/DAIC-WOZ project. This is the most widely used benchmark for multimodal depression and suicidality analysis from remote interviews. By not citing this, the authors ignore the primary standard against which all such models are compared.

DeVault D, Artstein R, Benn G, et al. "SimSensei Kiosk: A virtual human interviewer for healthcare decision support." Proceedings of the 2014 International Conference on Autonomous Agents and Multi-agent Systems (AAMAS). 2014; May: 1061-1068. Relevance: Establishes the standard pipeline (OpenFace, OpenSMILE, Text) for interview-based automated diagnosis.

Gratch J, Artstein R, Lucas G, et al. "The Distress Analysis Interview Corpus of human and computer interviews." In Proceedings of the Ninth International Conference on Language Resources and Evaluation (LREC'14), pages 3123–3128, Reykjavik, Iceland. European Language Resources Association (ELRA). Relevance: Describes the DAIC-WOZ dataset, which the authors should have used for external validation or transfer learning.

Acoustic Biomarkers for Suicide (Specific Prior Art)

The manuscript cites general depression acoustic papers but misses key studies specifically focused on suicidality (which is distinct from depression). The following papers explicitly differentiate "suicidal" voice from "depressed" voice, a distinction the authors struggle with.

Cummins N, Scherer S, Krajewski J, Schnieder S, Epps J, Quatieri TF. "A review of depression and suicide risk assessment using speech analysis." Speech Communication. 2015 July; 71: 10-49. Relevance: The seminal review in the field. It details specific features (jitter, shimmer, formant frequencies) related to suicide risk that the authors' study aims to replicate.

Min S, Kim D, Lee H, et al. "Acoustic Analysis of Speech for Screening for Suicide Risk: Machine Learning Classifiers for Between- and Within-Person Evaluation of Suicidality." JMIR Mental Health. 2023; 10: e44062. DOI: 10.2196/44062. Relevance: Directly addresses the "fluctuation" of SI (a limitation noted in the manuscript) by comparing within-person vs. between-person models.

Low DM, Bentley KH, Ghosh SS. "Automated assessment of psychiatric disorders using speech: A systematic review." Laryngoscope Investig Otolaryngol. 2020 Jan 31;5(1):96-116. doi: 10.1002/lio2.354. Relevance: A comprehensive review that catalogs the exact acoustic features (e.g., pause duration, speaking rate) the authors utilized, providing necessary context for their feature selection.

**Response**

We thank the reviewer for suggesting public dataset for external validation, the absence of an independent external dataset is a limitation of this study and have certainly limited the generalizability of the models. Currently, the lack of external validation was mainly because the public datasets are not directly aligned with our study design. Specifically, our task focuses on suicidal ideation within patients with MDD, whereas these public datasets are largely designed for depression detection/severity or other related tasks. In addition, our cohort consists of Chinese-speaking participants, and both linguistic and acoustic features may vary substantially across languages. Finally, our data were collected using a smartphone-based EMA protocol with multimodal features in a naturalistic setting, which differs from the structured interview settings and available modalities of the datasets.

We also thank the reviewer for identifying these relevant prior publications. In the revised manuscript, we incorporated the suggested references and expand the Introduction and Discussion accordingly. (Page25-26)

“Evidence from studies in other mental disorders further suggests that these multimodal approaches may be valuable for early detection and clinical risk prediction.[10, 11] Despite these advancements, multimodal analysis for suicidal ideation (SI) detection remains limited. A recent review found that only 3 out of 14 multimodal, speech-based studies focused specifically on SI detection.[12] While suicidal ideation typically occurs before suicidal behaviors, these methods have the potential to identify risk at an earlier stage, enhancing suicide prevention efforts.” (Page 5)

“This work also builds on early precedent showing that videotaped analysis of non-verbal behavior in depressed patients can yield clinically meaningful signals, as demonstrated by Ulrich in 1985.[36]” (Page 21)

“The importance of facial features such as AU12 and AU26 in our models is broadly consistent with prior work showing that depression-related signal is partly expressed through altered facial affect and reduced expressivity. Jiang et al.[46] found that happiness-related facial features were the most informative predictors in remission/non-response classification, with average and maximum happiness ranking highest and AU4 contributing negatively, supporting the relevance of affect-linked facial behavior in psychiatric assessment. Although the exact AUs differed, our findings similarly suggest that lower-face expressive actions may carry clinically meaningful information. Our multimodal findings are also aligned with Jiang et al. [47], who reported that facial, vocal, linguistic, and cardiovascular features extracted from remote interviews all contributed to mental health classification, with multimodal fusion outperforming most unimodal models. In particular, their findings of reduced facial expressivity, increased sadness in language, and relevant acoustic variation support the biological plausibility of our facial, linguistic, and acoustic predictors.

Feature importance of acoustic features such as Shimmer contributed to SI prediction in the model is also consistent with prior literature showing that acoustic biomarkers carry information relevant to psychiatric states, particularly depression-related alterations.” (Page 23-24)

“Therefore, while our method provides a practical foundation for examining associations between digital features and recent SI in clinical populations[49], future research should strive for closer alignment of assessment timing to enhance the accuracy of such associations.” (Page 24)

**Comment R2.7**

Recent State-of-the-Art (SOTA) Comparisons (2020-2025)

The authors use older NLP methods (LIWC). They fail to acknowledge that the field has shifted to Large Language Models (LLMs) and Deep Learning fusion.

Gao, Y., Fu, J., Guo, L., & Liu, H. (2025). “Leveraging Large Language Models for Spontaneous Speech-Based Suicide Risk Detection.” arXiv preprint arXiv:2507.00693. Relevance: Demonstrates that LLMs outperform the dictionary-based methods (LIWC) used by the authors. This is a critical "SOTA" comparison missing from their Discussion.

**Response**

We sincerely thank the reviewer for this insightful comment and for highlighting recent advances in LLM-based approaches. We agree that large language models have shown strong performance in speech- and text-based mental health prediction tasks, including suicide risk detection[7]. However, these approaches primarily focus on predictive performance and often rely on high-dimensional latent representations that are difficult to interpret in terms of clinically meaningful linguistic constructs.

In contrast, our study adopts LIWC as it provides theory-driven and psychologically validated linguistic categories that directly correspond to interpretable behavioral and affective dimensions. Since our objective is not only prediction but also the identification of linguistically grounded biomarkers, interpretability and construct validity are essential requirements.

While LLM-based methods may achieve higher predictive accuracy in some settings, they currently lack stable and standardized mappings to clinically interpretable psychological constructs, and their explanations often depend on post-hoc methods that may not reflect true model reasoning. Therefore, LIWC remains more suitable for our study’s goal of biomarker identification and interpretability-driven analysis.

We will revise the discussion to acknowledge LLM-based approaches and clarify the rationale for selecting LIWC in this context. The following are added:

“Although recent studies have reported strong performance of LLM-based approaches for suicide risk detection[38], LIWC remains preferable in this study due to its psychologically grounded and interpretable linguistic categories, which are suitable for biomarker identification” (Page22)

Ref[7]: Gao Y, , Fu, J., Guo, L., & Liu, H. Leveraging Large Language Models for Spontaneous Speech-Based Suicide Risk Detection. ArXiv. 2025.

**Comment R2.8**

Perform a Nested Cross-Validation (Outer loop for error estimation, Inner loop for feature selection/hyperparameter tuning) to ensure the feature selection step is not biasing the results. (The text mentions feature selection "within each cross-validation fold," which suggests they might be doing this correctly, but a clearer description of a Nested CV structure is needed)

**Response**

We agree that nested cross-validation is the preferred structure for reducing biase. However, our total sample size was modest (n=99) and the suicidality-related subgroup distribution was uneven. Prior work has shown that, in small-sample predictive modeling settings, cross-validation estimates inherently have large error bars and substantial instability[8]. We were therefore concerned that a full nested CV framework would further reduce the effective training data within each outer fold and make feature selection and hyperparameter tuning more unstable, particularly for the minority suicidality-related subgroup. We acknowledge this as a limitation and have clarified it in the revised manuscript as an exploratory result. (Page 27)

Ref[8]: G V. Cross-validation failure: Small sample sizes lead to large error bars. Neuroimage. 2018;180(Pt A):68-77. doi: 10.1016/j.neuroimage.2017.06.061.

**Comment R2.9**

Compare Features: In the Discussion, compare the specific significant features found (e.g., AU12, MFCCs) with those reported in Jiang et al. (2021) and Jiang et al. (2024) to discuss biological plausibility. In the Results, a breakdown of which features were most predictive and how many were used to achieve the highest performances is needed.

Also, there are hyperparameters that were selected outside of the CV training, and the method of selecting needs to be defined/addressed. For instance, in the pre-processing, how were the parameters like 0.7 (line#163) selected?

**Response**

Thank you for this suggestion. We revised the results to include the feature importance for some of the best models. Specifically, we now report that the potential effective predictors included facial (AU12, AU26), acoustic (shimmer), linguistic (negative emotion, time, informal), and questionnaire variables. We also compared our results with some of the previous work to discuss the biological plausibility. The detailed discussion is in Page 25.

For the confidence threshold of 0.7 was selected to retain only high-confidence face detections in OpenFace, where the confidence score reflects the reliability of facial landmark tracking[9]. Guidance from the OpenFace developers also indicates that thresholds around 0.7 are appropriate for filtering unreliable frames. In addition, similar threshold values (≈0.6–0.7) are commonly used in face detection pipelines to obtain more reliable detections[11].

In our case, 0.7 was chosen as a conservative operating point to prioritize data quality for downstream analysis. A brief sensitivity check (0.5–0.8) showed that lower thresholds introduced noisier detections, while higher thresholds reduced the number of usable frames without clear benefit. Therefore, 0.7 was selected as a practical balance. The has been clarified and the following text are added “To ensure data quality, based on a preliminary sensitivity analysis, a confidence threshold of 0.7 was applied, retaining only frames above the threshold.”

Ref [9]: Baltrusaitis, T., Zadeh, A., Lim, Y. C., & Morency, L. OpenFace 2.0: Facial Behavior Analysis Toolkit. 13th IEEE International Conference on Automatic Face & Gesture Recognition(FG 2018). 2018:59–66.

Ref[10]; Chancejia. How to get the confidence value? Online forum post GitHub. 2017;https://github.com/TadasBaltrusaitis/OpenFace/issues/270.

Ref[11]: Zhang K, Zhang, Z., Li, Z., & Qiao, Y. Joint face detection and alignment using multitask cascaded convolutional networks. IEEE Signal Processing Letters. 2016;23(10):1499–503. doi: https://doi.org/10.1109/lsp.2016.2603342.

**Comment R2.10**

5. Proofreading:

-Correct typos like "Appemdix" and ensure "P-values of the AUC" are calculated using appropriate tests for correlated ROC curves (e.g., DeLong).

-The Statistical analysis section is not very clear. For instance, what are the 3 groups mentioned (line#235)

-Line#138, Not clear how it mitigates data leakage?

-Lines#155,156 talk about moods and activities, but then talk about moods and not activities. Are the activities part dropped as a whole? If so, why?

-Through the paper, the discussion is about balanced data, but in lines 215,216 the imbalanced datasets are brought up, which makes it unnecessarily confusing.

**Response**

We thank the reviewer for the careful proofreading and for identifying these issues. We removed the inappropriate AUC P-values and revised the Statistical Analysis section for clarity. In addition, we have also added text to better explain how we mitigate data leakage and clarified that the whole recording captured is used.

**Comments from Reviewer 3**

**Comment R3.1**

Suicide prevention remains a major global health priority, and the integration of ecological momentary assessment (EMA) with machine learning represents a promising direction for scalable digital mental health solutions.

The manuscript is clearly written, ethically conducted, and aligned with the journal’s scope. The multimodal framework incorporating facial, acoustic, linguistic, and questionnaire-derived features collected via smartphone-based EMA is innovative and demonstrates feasibility in real-world settings. However, several methodological and translational issues limit the strength of the conclusions at this stage. I outline major and minor concerns below.

Major Concerns

1. Sample Size and High-Dimensional Feature Space

The study includes 99 participants (38 SI, 61 non-SI) with 1009 derived features. This results in a very high feature-to-sample ratio, raising substantial concerns regarding overfitting and model stability. Although ANOVA-based feature selection was performed within cross-validation folds, the dimensionality remains extremely large relative to sample size.

Please clarify:

Whether hyperparameter tuning and feature selection were fully nested within cross-validation.

Whether any stability analysis of selected features was performed across folds.

Whether confidence intervals for AUC and F1-score were calculated.

Without nested validation and stability assessment, performance estimates may be optimistic.

**Response**

We thank the reviewer for raising this important concern. We agree that the combination of a modest sample size increases the risk of overfitting and instability, and we have revised the manuscript to clarify the validation procedures. We clarified that feature selection was performed within the cross-validation procedure, rather than on the full dataset prior to model evaluation, in order to reduce the risk of information leakage. After feature selection, the number of features were reduced to a more manageable range, approximately 20–50 features depending on the model. We have added 95% confidence intervals for AUC and F1-score to better reflect the model performance estimates in this small-sample setting. In addition, to examine whether model findings were driven by unstable predictors, we added feature importance analyses and compared the relative contribution of modalities across models. (Page 18-19)

Regarding nested cross-validation, we agree that a fully nested framework is generally preferable for reducing optimism in performance estimation. However, given the modest total sample size and the uneven class distribution between the SI and non-SI groups, we were concerned that nested cross-validation design would further reduce the effective training data available within each outer fold. In this setting, this could make both feature selection and hyperparameter tuning even less stable, particularly for the minority SI group. Prior work has shown that, in small-sample predictive modeling settings, cross-validation estimates inherently have large error bars and substantial instability[8]. Due to the potential optimism, the findings would be better interpreted as exploratory and proof-of-concept rather than definitive. We have revised the manuscript accordingly to discuss more about the limitations and better contextualize the performance results.

Ref [8]: G V. Cross-validation failure: Small sample sizes lead to large error bars. Neuroimage. 2018;180(Pt A):68-77. doi: 10.1016/j.neuroimage.2017.06.061.

**Comment R3.2**

2. Absence of External Validation

All performance metrics are based on internal cross-validation. No external validation cohort was used. Given:

Single-site recruitment (Hong Kong),

Cantonese linguistic processing,

Cultural speech and affective expression patterns,

generalizability is uncertain.

The manuscript appropriately acknowledges this limitation, but the conclusions should be further tempered to emphasize the exploratory/proof-of-concept nature of the findings.

**Response**

We thank the reviewer for this important point. All reported performance metrics were derived from internal cross-validation only. Therefore, the findings should be interpreted as exploratory and proof-of-concept rather than generalizable clinical performance.

We considered publicly available datasets such as DAIC-WOZ, but they are not well matched to our study objective or protocol. Our task focuses specifically on suicidal ideation in patients with MDD, whereas these datasets are often designed for depression detection or severity estimation. In addition, our cohort consists of Chinese-speaking participants, and both linguistic and acoustic features are likely to vary across languages and cultural contexts. Our study also used a smartphone-based EMA protocol in a naturalistic setting, which differs substantially from the structured interview format and available modalities in these public datasets.

We have revised the manuscript to strengthen this limitation and emphasized that the exploratory nature of the findings. The following text have been added:

“In addition, the absence of external validation restricts the generalizability of the findings and raises concerns regarding their generalisability, the reported results should be interpreted as exploratory rather than as evidence of generalizable clinical performance.” (Page 25)

**Comment R3.3**

3. Binary Operationalization of Suicidal Ideation

Suicidal ideation was defined as H11 ≥ 1, collapsing passive death wishes, active ideation, and attempts into a single binary category. This may obscure clinically meaningful distinctions and introduces heterogeneity in the positive class.

Consider discussing:

Why ordinal or multiclass modeling was not attempted.

The distribution of H11 severity levels within the SI group.

Whether performance differs for higher-severity SI.

**Response**

We agree that binary operationalization may obscure clinically meaningful distinctions. We used a binary definition because the distribution of H11 severity levels was imbalanced, making ordinal or multiclass modeling less feasible in this sample. Higher-severity cases were rare, with only one participant in the suicide-attempt category. We have clarified this limitation in the manuscript and future studies with larger samples would examine multiclass prediction of suicidality severity. The following text have been added:

“In addition, suicidal ideation was operated as a binary outcome, which may have obscured clinically differences in severity. This choice was primarily driven by the imbalanced distribution of H11 severity levels in our sample, including relatively few higher-severity cases.” (Page 25)

**Comment R3.4**

4. Incremental Value of Multimodal Features

HADS-A, HADS-D, and TAS subscales show strong group differences. It is unclear how much predictive value multimodal digital features add beyond questionnaire measures.

An ablation analysis would substantially strengthen the manuscript:

Questionnaire-only model

Multimodal digital features only

Combined model

This would clarify whether digital phenotyping provides meaningful incremental utility.

**Response**

We thank the reviewer for this important suggestion. To assess incremental value, we included baseline models using demographic and questionnaire features only and compared it against a model incorporating demographic, questionnaire, and multimodal digital features. We have revised the manuscript to include the comparison in the results section and to assess the incremental contribution of multimodal features. The added texts are as follow:

“To assess whether multimodal digital features added predictive value beyond conventional clinical information, we compared models trained on demographic and questionnaire variables (Table 6) alone with models trained on broader feature sets including multimodal data. The best performance was obtained using the combined demographic, questionnaire, and multimodal with CatBoost achieving an AUC of 0.79, F1 score of 0.73, and accuracy of 0.79. In comparison, the best baseline model using demographic and questionnaire features alone achieved lower performance. The difference between the baseline model and multimodal models is 5-8% in term of AUC and F1-score, greater improvement is seen across accuracy, specificity, PPV and NPV. This pattern indicates that multimodal features provide potential incrementative predictive information beyond standard self-report and demographic measures. Importantly, the highest performance was not obtained from multimodal data alone, but from their integration with conventional variables, suggesting that multimodal feature could be useful as an additive to questionnaire-based assessment.

An additional pattern was observed across model types. Baseline models using demographic and questionnaire features tended to perform relatively well with simpler models such as logistic regression and random forest, whereas models incorporating multimodal features usually achieved stronger performance with more flexible approaches such as ANN and CatBoost. Although this pattern should be interpreted cautiously, it may suggest that the relationship between multimodal features and SI is more complex and potentially more nonlinear than questionnaire and demographic features, requiring models that can better capture feature interactions and higher-order structure.” (Page 19-21)

**Comment R3.5**

5. Clinical Utility and Sensitivity

The best-performing model (CatBoost) shows:

AUC = 0.79

Sensitivity = 0.53

Specificity = 0.89

While specificity is strong, sensitivity is modest, meaning nearly half of SI cases would be missed. For suicide screening, high sensitivity is typically prioritized.

Please discuss:

Intended clinical deployment scenario (screening vs. rule-in tool)

Threshold optimization strategy

Whether recall-focused optimization was considered

**Response**

We thank the reviewer for this important point. In the revised analysis, we optimized the operating threshold using F1-score optimization. This choice was motivated by the fact that our task is an imbalanced binary classification problem[12], and high precision and recall are both important in clinical settings. In addition, prior work has shown that, for classifiers producing real-valued outputs, there is a principled relationship between the maximum achievable F1 value and the decision threshold that attains it[12]. At the same time, we acknowledge that no single performance measure can capture every desirable property, alternative thresholding approaches may also be preferable in clinical setting. The following text has been added:

“Model selection was mainly based on the F1-score and AUC, thresholds are calibrated using an F1-prioritized criterion” (Page 10)

Ref [12]: Lipton, Z. C., Elkan, C., & Naryanaswamy, B. Optimal Thresholding of Classifiers to Maximize F1 Measure. Mach Learn Knowl Discov Databases. 2014;8725:225-39. doi: 10.1007/978-3-662-44851-9_15.

**Comment R3.6**

6. Model Interpretability

Interpretability is essential in psychiatric AI. The manuscript would benefit from:

Feature importance analysis

SHAP or similar explainability methods

Discussion of clinically interpretable signals

Understanding which modalities contribute most would improve translational relevance.

**Response**

To improve model interpretability, we added a feature importance analysis in the results section and compared the feature importance across models to confirm the consistency of the findings. This analysis also allowed us to examine which modalities contributed most strongly to prediction and which features contributed the most towards the prediction. In addition, we expanded the discussion to compare the features and modalities to prior literature, in order to assess the clinical plausibility and its consistency with existing knowledge. The added text are as follow:

“Based on the feature importance of the best performing models, the predictive information was distributed across multiple modalities. In the CatBoost model, the strongest predictors included facial, acoustic, linguistic variables, and several HADS items, with AU26, Shimmer, and AU12 ranking highest.

In the Naive Bayes model, questionnaire variables were relatively more prominent, but multimodal variables also showed relevant importance, including linguistic variable “informal”, Shimmer and AU26.

Facial features such as AU12 and AU26, linguistic features including negative emotion, time, and informality, as well as the acoustic variable shimmer, consistently showed relative importance across the models. Greater contribution by facial and linguistic features are seen over acoustic features. Overall, the findings support a multimodal contribution to prediction, with the noticeable multimodal features.” (Page 18-19)

“The importance of facial features such as AU12 and AU26 in our models is broadly consistent with prior work showing that depression-related signal is partly expressed through altered facial affect and reduced expressivity. Jiang et al.[47] found that happiness-related facial features were the most informative predictors in remission/non-response classification, with average and maximum happiness ranking highest and AU4 contributing negatively, supporting the relevance of affect-linked facial behavior in psychiatric assessment. Although the exact AUs differed, our findings similarly suggest that lower-face expressive actions may carry clinically meaningful information. Our multimodal findings are also aligned with Jiang et al.[48], who reported that facial, vocal, linguistic, and cardiovascular features extracted from remote interviews all contributed to mental health classification, with multimodal fusion outperforming most unimodal models. In particular, their findings of reduced facial expressivity, increased sadness in language, and relevant acoustic variation support the biological plausibility of our facial, linguistic, and acoustic predictors.

Feature importance of acoustic features such as Shimmer contributed to SI prediction in the model is also consistent with prior literature showing that acoustic biomarkers carry information relevant to psychiatric states, particularly depression-related alterations.” (page 23-24)

**Comment R3.7**

7. Data Availability and PLOS Policy

The Data Availability Statement indicates that data are available upon reasonable request. PLOS requires that all data underlying the findings be fully available without restriction, except in rare justified circumstances.

If privacy restrictions prevent full public release, please:

Specify the exact nature of restrictions,

Indicate whether de-identified feature matrices (without raw video/audio) can be deposited,

Clarify repository plans.

This is important for reproducibility.

**Response**

We thank the reviewer for the suggestion. The dataset supporting this study is not currently available due to the ongoing nature of the project. We will share the full data upon reasonable request, in accordance with institutional and journal policies. The revised data availability statement is as follows:

“Data can be requested to the following: The institutional contact: Li Chiu Kong Family Sleep Assessment Unit sleepresearch@cuhk.edu.hk

Full citation of the data: Li Chiu Kong Family Sleep Assessment Unit. DMoMo data. The Chinese University of Hong Kong: Hong Kong, China; 2025 [cited 2025 Dec 18].

Available from: sleepresearch@cuhk.edu.hk.

The third-party organization - ethics committee: You can email the Joint CUHK-NTEC Clinical Research Ethics Committee at crec@cuhk.edu.hk for inquiries about our clinical research ethics.” (Page 28)

**Comment R3.8**

Minor Comments

Some grammatical refinements are needed (“This study is a few of its kind”).

Claims such as “great capability” should be softened.

Consider adding calibration curves and reporting 95% confidence intervals for AUC.

Clarify the rationale for generating 980 derived features and whether dimensionality reduction alternatives were considered.

**Response**

We thank the reviewer for these helpful suggestions. In the revised manuscript, we have corrected grammatical issues throughout the text, including phrasing such as “This study is a few of its kind,” and we have softened expressions to ensure a more cautious and appropriate tone. We will also report 95% confidence intervals for AUC to better interpret the model performance. ANOVA F-value was chosen because it is interpretable, and appropriate for supervised filtering in a relatively small-sample, high-dimensional setting.
