# Supplementary material for: Identification of suicidal ideation in major depressive disorder: A machine learning approach with multimodal digital features
Source: PLOS Digit Health. 2026 Aug 27;5(8):e0001615. doi: 10.1371/journal.pdig.0001615 (PMC13521398; doi:10.1371/journal.pdig.0001615)
Supplement: Supplementary file 4 [file pdig.0001615.s005.docx]

Dear Editor and Reviewers,

We would like to express our gratitude for your comments and suggestions. The comments helped us to substantially improve our paper. We have carefully looked through the concerns raised by the reviewers, and we hope we have sufficiently addressed them in this revision. In this document, we discuss our responses to the comments in detail. Thank you for your precious time and valuable suggestions.

Yours sincerely,

Authors

**Comments from Reviewer 1**

**Comment R1.1**

I thank the authors for their efforts in revising their manuscript. My comments have been sufficiently	addressed.

**Response**

Thank you for your previous comments and suggestions.

**Comments from Reviewer 3**

**Comment R3.1**

The revised manuscript is substantially improved compared with the previous version, particularly in the clarification of the study objectives, the discussion of limitations, the addition of baseline comparisons, and the expanded interpretation of the multimodal features.

The topic is clinically important, and the use of smartphone-based ecological momentary assessment combined with facial, acoustic, linguistic, and questionnaire-derived features represents an interesting and potentially useful direction for digital mental health research. I also appreciate the authors' efforts to frame the findings more cautiously as exploratory rather than presenting them as evidence of clinical readiness.

That said, several limitations remain and should continue to be emphasized throughout the manuscript.

First, the study is based on a relatively small cohort (N=99) with a high-dimensional feature space (>1000 features). Although feature selection was performed within the cross-validation framework, the risk of overfitting remains substantial. The absence of nested cross-validation and external validation makes it difficult to determine how well the reported performance would generalize to independent populations.

**Response**

We thank the reviewer for raising this point. We agree that the risk of overfitting, given the small cohort and the high dimensionality of the feature space, is a key consideration in interpreting our findings despite having feature selection. We also agree that the nested cross-validation and external validation would limit the generalizability of this study and future validation is needed before any conclusion on clinical utility could be drawn.

We have revised the manuscript to strengthen this limitation and emphasized that the exploratory nature of the findings. The following text have been added:

“Third, the sample size for both SI and non-SI MDD subjects were limited and with the absence of fully nested cross-validation, this could potentially constrain the robustness of model training and reducing the reliability of the validation results.”(page 24)

“Moreover, the high dimensionality of the feature space relative to the sample size still poses a risk of overfitting; although feature selection was conducted within the cross-validation framework to mitigate this, residual overfitting cannot be fully excluded.” (Page 24-25)

“Most importantly, the absence of external validation in independent cohorts remains the principal limitation of this study and fundamentally restricts the generalizability of the findings. As the models were developed within a single, relatively small cohort, their performance in other populations, settings, or languages cannot be ensured. The results should therefore be regarded as exploratory and hypothesis-generating rather than as evidence of generalizable clinical performance. Validation in larger, independent cohorts is essential before any conclusions regarding clinical utility or real-world deployment can be drawn.”(page 25)

**Comment R3.2**

Second, while the newly added baseline comparison is valuable, the improvement achieved by incorporating multimodal features appears modest. This finding is still important, but it suggests that the multimodal signals should be interpreted as providing incremental rather than transformative predictive value.

**Response**

We thank the reviewer and have revised the manuscript to frame the multimodal features as providing incremental rather than transformative predictive value. We also stated that this incremental contribution should be interpreted under the exploratory, proof-of-concept nature of the work.

We have revised the manuscript, the following text have been added:

“Although incorporating multimodal digital features improved predictive performance relative to the baseline derived from demographic and questionnaire variables alone, the improvement was modest (approximately 5–8% in AUC and F1-score). This could only indicate that facial, acoustic, and linguistic signals provide incremental rather than transformative predictive value. The best performance came not from multimodal data alone but from its integration with conventional variables, suggesting these features should be understood as an additive to questionnaire-based assessment.” (Page 22)

**Comment R3.3**

Third, the manuscript would benefit from additional discussion regarding the stability and reproducibility of the selected features across folds. Given the exploratory nature of the work, understanding whether similar features consistently emerge would strengthen confidence in the reported digital biomarkers.

**Response**

We thank the reviewer for this constructive suggestion. We have added a part in results reporting how consistently each feature was selected across the five cross-validation folds. This convergence across folds and across two distinct models strengthens the confidence of candidate digital biomarkers.

The revised texts are as follows:

“To assess reproducibility, we examined the consistency of multimodal feature selection across the five cross-validation folds. Several features recurred across folds in both models. In CatBoost, linguistic variable “informal” ranked highly in all 5 folds, with AU26 and Shimmer in 4 folds, and AU12 and linguistic variable “time” in 3 folds. The Naïve Bayes model showed a similar pattern, with linguistic variable “informal” selected in all 5 folds, Shimmer in 4 folds, and AU26, AU12, and linguistic variable “time” in 3 folds. The convergence of the same facial (AU12, AU26), acoustic (shimmer), and linguistic (informality, time) features across folds and across two distinct models indicates that these multimodal signals were selected in a stable and reproducible manner. Overall, the findings support a multimodal contribution to prediction, with the noticeable multimodal features.” (Page 19)

**Comment R3.4**

Finally, the lack of external validation remains the principal limitation of the study. I agree with the authors that publicly available datasets may not perfectly match the study design, language, or target population. Nevertheless, future validation in independent cohorts will be essential before any claims regarding clinical utility or deployment can be supported.

**Response**

We thank the reviewer and agree that future validation is essential before any claims regarding clinical utility could be supported. We have revised the discussion sesction to further emphasize this limitation, the added text are as follows:

“Most importantly, the absence of external validation in independent cohorts remains the principal limitation of this study and fundamentally restricts the generalizability of the findings. As the models were developed within a single, relatively small cohort, their performance in other populations, settings, or languages cannot be ensured. The results should therefore be regarded as exploratory and hypothesis-generating rather than as evidence of generalizable clinical performance. Validation in larger, independent cohorts is essential before any conclusions regarding clinical utility or real-world deployment can be drawn.” (Page 25)

“Additionally, this study is exploratory and serves as a proof-of-concept of use of multimodal features in suicide detection and identifies key multimodal features that may be relevant to the detection of suicidality. Further validation and investigation is needed before clinical utilities could be ensured.” (page 26)
