# Supplementary material for: Identification of suicidal ideation in major depressive disorder: A machine learning approach with multimodal digital features
Source: PLOS Digit Health. 2026 Aug 27;5(8):e0001615. doi: 10.1371/journal.pdig.0001615 (PMC13521398; doi:10.1371/journal.pdig.0001615)
Supplement: Peer review history

# Peer review: Decision Letter 0

**DOI:** 10.1371/journal.pdig.0001615.r001

**Authors:** Henry Horng-Shing Lu, Xiaoxia Wang

3 Mar 2026

Response to Reviewers Revised Manuscript with Track Changes Manuscript **Journal Requirements:**

1. Please provide a detailed online Financial Disclosure statement. This is published with the article. It must therefore be completed in full sentences and contain the exact wording you wish to be published.

a) State the initials, alongside each funding source, of each author to receive each grant. For example: “This work was supported by the National Institutes of Health (####### to AM; ###### to CJ) and the National Science Foundation (###### to AM).”

b) State what role the funders took in the study. If the funders had no role in your study, please state: “The funders had no role in study design, data collection and analysis, decision to publish, or preparation of the manuscript.”

For more information, please go to our submission guidelines:

https://journals.plos.org/digitalhealth/s/submission-guidelines#loc-financial-disclosure-statement

2. Please ensure that the funders and grant numbers match between the Financial Disclosure field and the Funding Information tab in your submission form. Note that the funders must be provided in the same order in both places as well.

3. Please send a completed 'Competing Interests' statement, including any COIs declared by your co-authors. Please declare all competing interests beginning with the statement “I have read the journal's policy and the authors of this manuscript have the following competing interests:”

For further assistance, you may go to: https://journals.plos.org/digitalhealth/s/competing-interests

4. In the online submission form, you indicated that "The data that support the findings of this study are available from the corresponding author upon reasonable request.".

All PLOS journals now require all data underlying the findings described in their manuscript to be freely available to other researchers, either

a) In a public repository,

b) Within the manuscript itself, or

d) Uploaded as supplementary information.

This policy applies to all data except where public deposition would breach compliance with the protocol approved by your research ethics board. If your data cannot be made publicly available for ethical or legal reasons (e.g., public availability would compromise patient privacy), please explain your reasons by return email and your exemption request will be escalated to the editor for approval. Your exemption request will be handled independently and will not hold up the peer review process, but will need to be resolved should your manuscript be accepted for publication. One of the Editorial team will then be in touch if there are any issues.

For further assistance, you may go to: http://journals.plos.org/digitalhealth/s/data-availability

5. If the reviewer comments include a recommendation to cite specific previously published works, please review and evaluate these publications to determine whether they are relevant and should be cited. There is no requirement to cite these works unless the editor has indicated otherwise.

**Additional Editor Comments (if provided):** **Reviewers' Comments:**

**Comments to the Author**

1. Does this manuscript meet PLOS Digital Health’s publication criteria?

Reviewer #1: Yes

Reviewer #2: No

Reviewer #3: Partly

**********

2. Has the statistical analysis been performed appropriately and rigorously?

Reviewer #1: Yes

Reviewer #2: No

Reviewer #3: No

**********

3. Have the authors made all data underlying the findings in their manuscript fully available (please refer to the Data Availability Statement at the start of the manuscript PDF file)?

The PLOS Data policy

Reviewer #1: No

Reviewer #2: No

Reviewer #3: No

**********

4. Is the manuscript presented in an intelligible fashion and written in standard English?

Reviewer #1: Yes

Reviewer #2: Yes

Reviewer #3: Yes

**********

Reviewer #1: I wish to thank the authors for their valuable contribution to the important field of suicide prevention that continues to elude researchers and health officials. I further wish to highlight the quality and expertise found in the submitted work. The multi-modal assessment is a clear strong point that brings together many known risk factors for suicidal ideation, and I encourage the collaborators to distribute the underlying software widely.

In the following I provide comments that are mainly aimed at clarifying some aspects of the manuscript.

Major Comments

1) The larger question that will decide if such digital modality EMA are worth using in research and practice is if we can predict suicidal ideation more accurately than if we simply ask patients to self-report their current suicidal ideation. As I understand it, suicidal ideation was not assessed during the EMAs and this comparison not the goal of this study. It would be important to discuss the reasoning for these choices in the paper more clearly. This can maybe also be addressed by outlining more clearly how the authors view the role of this technology in patient care of the future.

2) The Introduction is quite short and could benefit from discussing the cited literature in greater detail, to place it more clearly in the greater research context. Also the question of ‘why are we looking at suicidal ideation instead of suicidal behavior’ is currently not addressed.

3) The data availability statement is currently insufficient. Please discuss in more detail what data could be potentially be shared and how. While the recorded videos naturally cannot be shared, maybe facial action units or aggregate measures thereof be shared? Some clinical information and timestamps may need to be perturbed or fully removed to ensure anonymity.

4) 350 – 353: There is some grammatical confusion here, which should be clarified. The word “me” is a first-person pronoun just as much as “I” and therefore “I am sad” and “They upset me” both contain a first-person pronoun. It is important to confirm exactly how this was coded in LWIC. If “They upset me” does not contain a first-person pronoun in Cantonese, then please describe this.

5) Missing data is reported to be 0.05% (line 197), but it is unclear what that means. Does this mean that we have 99.95% rate of responding to EMA prompts? 99 participants x 7 days x 4 EMAs per day = 2772 responses. 0.05% missing would mean only one response (rounded) is missing. Please clarify if this is accurate in the text as it would constitute a phenomenal compliance rate. As feasibility of such technology is an important insight produced by this study, this should also be brought up in the discussion.

6) This paper does not require p-values. Table 1 should never contain p-values, as they do not aid interpretation and do not test a hypothesis; for reference see Cole et al. (2025)Ref. This extends in this paper to Table 2 as well. Further, I would also advise against reporting the p-value of the AUC. While not mathematically incorrect, it does not aid interpretation and is certainly unusual. In Tables 3 & 4 it could be argued that p-values make sense as the differences here are less clearly established, however in that case the p-values need to be corrected for multiple comparisons. Alternatively, it could be explained why there was not correction for multiple comparisons.

Ref.: Cole, J. B., Knack, S. K. S., & Driver, B. E. (2025). The value of P-values in "Table 1". The American journal of emergency medicine, 100, 182–186. Advance online publication. https://doi.org/10.1016/j.ajem.2025.12.003

7) The feature selection aspect feels underdeveloped. A few more words on the reasoning why it was done or if there were patterns in the optimal amount of features would be welcome. Further, it would be of high interest to learn which features were the strongest predictors. This could very valuable to follow-up research.

The following minor comments do not require a point-by-point response. In general, there are minor language issues regarding word choice and spelling, of which I will just highlight some that caught my eye. I recommend another close read or attentive copy editing.

Minor comments

-For Prof. Joey Chan, the middle names are only given as initials, while the others are spelled out.

-The section Machine Learning algorithms should mention that the full specification and results of the grid search are in the supplementary material.

-In Study Design (Line 118) it is mentioned that the study is ongoing. Does this mean that you are still collecting data? If so, it would be good to highlight that this is an interim analysis.

-In Table 1 there is inconsistency in reporting. Education, employment status and income category should have the same format as sex and marital status (N, %).

-The sentence from line 322 to 323 is phrased too strongly, especially as there is not yet any external validation.

Minor language comments

-Line 79: should be ‘accounts’ instead of ‘account’.

-Line 81: ‘adapted’ is likely meant to be ‘adopted’

-Line 101 – 105: ‘machine learning’ is repeated in the sentence.

-Line 207 & 211: ‘Appendix’ is misspelt as ‘Appemdix’.

-Line319: ‘a few of its kind’ is likely meant to be ‘the first of its kind’.

Reviewer #2: This manuscript presents a machine learning analysis to detect suicidal ideation (SI) in patients with Major Depressive Disorder (MDD) using multimodal data (facial, acoustic, linguistic) collected via a smartphone app (D-MOMO). While the objective is clinically significant and the ecological momentary assessment (EMA) approach is modern and relevant, the manuscript suffers from critical data inconsistencies, risks of overfitting due to high dimensionality vs. small sample size, and a lack of external validation. Furthermore, the work appears to be a secondary analysis of a dataset previously published by the same group without explicit clarification, raising questions about data slicing. Additionally, the article fails to acknowledge or cite a significant body of directly relevant prior work. The described approach to multimodal analysis (facial, vocal, linguistic) of remote interviews for mental health assessment is portrayed as novel, but this ignores significant published literature establishing very similar pipelines. The omission of these key references misrepresents the state of the art.

Here are some more detailed comments:

A. Problem Formulation, Data, and Techniques

1. Data Inconsistency and Integrity. There is a numerical inconsistency between the Abstract and the Results section that undermines confidence in the reported statistics. The abstract: Reports 31 patients with SI and 61 without SI (Total N = 92). The Results (and Table 1): Reports 38 patients with SI and 61 without SI (Total N = 99). It is unclear which dataset was used for the training and testing. If the abstract numbers are from a previous iteration, the abstract was not updated. If the results numbers are correct, the abstract is factually wrong. This must be resolved.

2. Overfitting risk. The study utilizes 99 subjects (at best) but extracts 1009 features. Even with the reported feature selection (ANOVA F-value), the ratio of observations to predictors is extremely low. While the authors use cross-validation (5-fold) and feature selection inside the fold (which is good practice to avoid leakage), the high dimensionality of the initial pool increases the "degrees of freedom" for finding spurious correlations. The effective sample size for the minority class (SI) is only ~38. In such small N studies, performance metrics like AUC often exhibit high variance. The reported AUC of 0.79 might not generalize to new data.

Also, investigating model performance using only features from survey and demographics to predict the H11 label (without the multi-modal data/features) would provide a baseline to compare and decide the contribution of multimodal data and to see how/if it improves the performance.

3. Label Definition and Temporal Mismatch. The target label (SI) is defined by a single item (H11) from the HDRS interview conducted at baseline. The features are collected via EMA over the following 7 days. Suicidal ideation is known to be transient and fluctuating. Labeling a full week of digital data based on a single "snapshot" interview conducted previously introduces significant label noise. A patient might have had SI during the interview but not during the app usage, or vice versa. The authors acknowledge this in limitations, but it fundamentally weakens the supervised learning setup. Moreover, it would be somewhat more reliable if this single item were collected after the 7 days of daily interactions/data collection, since it asks about thoughts from the last week.

4. Validation issues and missed opportunities on public data. The study relies entirely on internal 5-fold cross-validation. Given the small sample size and high feature count, internal CV is prone to optimistic bias. Without an independent hold-out set, the claim of "robust" performance is unsupported. The use of Mann-Whitney tests to compare AUCs across models on the same dataset (correlated samples) is often statistically underpowered or inappropriate compared to tests like DeLong’s test. The study relies exclusively on a small, private internal dataset (N=99?). The authors failed to leverage (through transfer learning) or benchmark against standard public datasets used in this domain.

Relevant Public Databases (Missing from Study):

DAIC-WOZ (Distress Analysis Interview Corpus - Wizard of Oz): This is the "gold standard" public dataset for multimodal depression detection, containing audio, video, and text of clinical interviews. The authors should have used this for transfer learning or as an external validation set.

EATD-Corpus (Emotional Audio-Textual Depression Corpus): A dataset specifically for depression detection using audio and text, highly relevant for their acoustic/linguistic analysis.

MODMA (Multi-modal Open Dataset for Mental-disorder Analysis): Includes EEG and audio from depressed patients, relevant for cross-modal comparisons.

B. Novelty issues and lack of appropriate citations and framing in terms of the existing literature.

1. The novelty of this work is low to moderate. The specific application of these multimodal features to Suicidal Ideation (SI) appears to be a new analysis. However, the data collection platform (D-MOMO), the study protocol, the participant pool (Hong Kong Chinese MDD patients), and the feature extraction pipeline (OpenFace, OpenSMILE, LIWC) have been previously published by this research group. This might be interpreted as salami-slicing of a dataset previously used to predict MDD diagnosis and remission status. The authors should explicitly state if this is a secondary analysis of the same cohort, and what is new in this approach.

2. Relevant Prior Publications are not cited. For instance, I'm very surprised that the authors missed the following:

The dataset and methods (D-MOMO, 7-day EMA, same features) appear identical to: Chen J, Chan NY, Li CT, Chan JWY, Liu Y, Li SX, et al. "Multimodal digital assessment of depression with actigraphy and app in Hong Kong Chinese." Translational Psychiatry. 2024;14(1):1-8. doi: 10.1038/s41398-024-02873-4. In the Chen et al. (2024) paper, the same group used the D-MOMO app on 61 MDD subjects and 47 controls to detect MDD. The current manuscript appears to slightly expand the recruitment (N=99) or combine groups to focus on the SI sub-question.

The following background articles and previous work predate this work and should be reviewed and cited:

The "Gold Standard" Dataset & Protocols (Missing Benchmarks)

The authors claim their multimodal video approach is novel, yet they fail to cite the SimSensei Kiosk/DAIC-WOZ project. This is the most widely used benchmark for multimodal depression and suicidality analysis from remote interviews. By not citing this, the authors ignore the primary standard against which all such models are compared.

DeVault D, Artstein R, Benn G, et al. "SimSensei Kiosk: A virtual human interviewer for healthcare decision support." Proceedings of the 2014 International Conference on Autonomous Agents and Multi-agent Systems (AAMAS). 2014; May: 1061-1068. Relevance: Establishes the standard pipeline (OpenFace, OpenSMILE, Text) for interview-based automated diagnosis.

Gratch J, Artstein R, Lucas G, et al. "The Distress Analysis Interview Corpus of human and computer interviews." In Proceedings of the Ninth International Conference on Language Resources and Evaluation (LREC'14), pages 3123–3128, Reykjavik, Iceland. European Language Resources Association (ELRA). Relevance: Describes the DAIC-WOZ dataset, which the authors should have used for external validation or transfer learning.

Acoustic Biomarkers for Suicide (Specific Prior Art)

The manuscript cites general depression acoustic papers but misses key studies specifically focused on suicidality (which is distinct from depression). The following papers explicitly differentiate "suicidal" voice from "depressed" voice, a distinction the authors struggle with.

Cummins N, Scherer S, Krajewski J, Schnieder S, Epps J, Quatieri TF. "A review of depression and suicide risk assessment using speech analysis." Speech Communication. 2015 July; 71: 10-49. Relevance: The seminal review in the field. It details specific features (jitter, shimmer, formant frequencies) related to suicide risk that the authors' study aims to replicate.

Min S, Kim D, Lee H, et al. "Acoustic Analysis of Speech for Screening for Suicide Risk: Machine Learning Classifiers for Between- and Within-Person Evaluation of Suicidality." JMIR Mental Health. 2023; 10: e44062. DOI: 10.2196/44062. Relevance: Directly addresses the "fluctuation" of SI (a limitation noted in the manuscript) by comparing within-person vs. between-person models.

Low DM, Bentley KH, Ghosh SS. "Automated assessment of psychiatric disorders using speech: A systematic review." Laryngoscope Investig Otolaryngol. 2020 Jan 31;5(1):96-116. doi: 10.1002/lio2.354. Relevance: A comprehensive review that catalogs the exact acoustic features (e.g., pause duration, speaking rate) the authors utilized, providing necessary context for their feature selection.

Recent State-of-the-Art (SOTA) Comparisons (2020-2025)

The authors use older NLP methods (LIWC). They fail to acknowledge that the field has shifted to Large Language Models (LLMs) and Deep Learning fusion.

Gao, Y., Fu, J., Guo, L., & Liu, H. (2025). “Leveraging Large Language Models for Spontaneous Speech-Based Suicide Risk Detection.” arXiv preprint arXiv:2507.00693. Relevance: Demonstrates that LLMs outperform the dictionary-based methods (LIWC) used by the authors. This is a critical "SOTA" comparison missing from their Discussion.

Li Y, et al. "Multimodal Speech and Text Models to Detect Suicidal Risks in Adolescents." medRxiv. 2025 August 5. DOI: 10.1101/2025.07.31.25332367. Relevance: A very recent study with a larger sample (N=600) using similar multimodal fusion. It highlights the inadequacy of the authors' small sample size (N=99).

Jiang Z, Luskus M, Seyedi S, Griner EL, Rad AB, Clifford GD, Boazak M, Cotes RO. Utilizing computer vision for facial behavior analysis in schizophrenia studies: A systematic review. PLoS One 2022 April 8; 17 (4): p. E0266828.2. Relevance: key review article addressing the same pipelines described in the submitted article.

Jordan E, Terrisse R, Lucarini V, Alrahabi M, Krebs MO, Desclés J, Lemey C. Speech Emotion Recognition in Mental Health: Systematic Review of Voice-Based Applications. JMIR Ment Health. 2025 Sep 30;12:e74260. doi: 10.2196/74260.

Han MM, Li XY, Yi XY, Zheng YS, Xia WL, Liu YF, Wang QX. Automatic recognition of depression based on audio and video: A review. World J Psychiatry. 2024 Feb 19;14(2):225-233. doi: 10.5498/wjp.v14.i2.225.

Min, K., Yoon, J., Kang, M. et al. Detecting depression on video logs using audiovisual features. Humanit Soc Sci Commun 10, 788 (2023). https://doi.org/10.1057/s41599-023-02313-6

Lee T, Baek S, Lee J, Chung ES, Yun K, Kim TS, Oh J. A Deep Learning Driven Simulation Analysis of the Emotional Profiles of Depression Based on Facial Expression Dynamics. Clin Psychopharmacol Neurosci. 2024 Feb 29;22(1):87-94. doi: 10.9758/cpn.23.1059. Epub 2023 Jun 8.

Ulrich G, Harms K. A video analysis of the non-verbal behaviour of depressed patients before and after treatment. J Affect Disord. 1985 Jul;9(1):63-7. doi: 10.1016/0165-0327(85)90011-4. Relevance: This is one of the earliest works - it's important to credit the precedents on this topic.

Jiang Z, Harati S, Crowell A, Mayberg HS, Nemati S, Clifford GD. Classifying Major Depressive Disorder and Response to Deep Brain Stimulation Over Time by Analyzing Facial Expressions. IEEE Trans Biomed Eng. 2021 Feb;68(2):664-672. doi: 10.1109/TBME.2020.3010472. Epub 2021 Jan 21.

Cotes RO, Boazak M, Griner E, Jiang Z, Kim B, Bremer W, Seyedi S, Bahrami Rad A, Clifford GD. Multimodal Assessment of Schizophrenia and Depression Utilizing Video, Acoustic, Locomotor, Electroencephalographic, and Heart Rate Technology: Protocol for an Observational Study. JMIR Res Protoc. 2022 Jul 13;11(7):e36417. doi: 10.2196/36417.

Jiang Z, Seyedi S, Griner E, Abbasi A, Rad AB, Kwon H, Cotes RO, Clifford GD. Multimodal Mental Health Digital Biomarker Analysis From Remote Interviews Using Facial, Vocal, Linguistic, and Cardiovascular Patterns. IEEE J Biomed Health Inform. 2024 Mar;28(3):1680-1691. doi: 10.1109/JBHI.2024.3352075. Epub 2024 Mar 6.

Mu X, Bibars M, Seyedi S, Zheng I, Jiang Z, Chen L, Omofojoye B, Hershenberg R, Levey AI, Clifford GD, Dodge HH, Kwon H. Feasibility of Detecting Cognitive Impairment and Psychological Well-being among Older Adults Using Facial, Acoustic, Linguistic, and Cardiovascular Patterns Derived from Remote Conversations. Machine Learning: Health 2025 November 27.

Ahmad F, Abbasi A, Li J, Dobolyi DG, Netemeyer RG, Clifford GD, Chen H. A deep learning architecture for psychometric natural language processing. ACM Transactions on Information Systems (TOIS) 2020 February 5; 38 (1): pp. 1-29.

Qin R, Cook R, Yang K, Abbasi A, Dobolyi D, Seyedi S, Griner E, Kwon H, Cotes R, Jiang Z, Clifford GD. Language Models for Online Depression Detection: A Review and Benchmark Analysis on Remote Interviews. ACM Transactions on Management Information Systems 2025 March 13; 16 (2): pp. 1-35. DOI: 10.1145/3673906.

4. Suggested Next Steps for Improvement

Conduct a Thorough Literature Review: The Introduction and Discussion must be rewritten to include the 8 articles listed above. The authors must explicitly state how their work differs from or builds upon earlier works in multimodal voice and video for psychiatric assessment.

Fix the Data Discrepancy: Immediately resolve the N=92 vs N=99 conflict between the Abstract and Results.

Clarify Data Origin: Explicitly state if this is a secondary analysis of the cohort from Chen et al. (2024). Transparency regarding the sample overlap is required.

Perform a Nested Cross-Validation (Outer loop for error estimation, Inner loop for feature selection/hyperparameter tuning) to ensure the feature selection step is not biasing the results. (The text mentions feature selection "within each cross-validation fold," which suggests they might be doing this correctly, but a clearer description of a Nested CV structure is needed)

Perform External Validation: The authors must attempt to validate their model on a public dataset like DAIC-WOZ. If their model (trained on Cantonese speakers) cannot be directly applied to English datasets, they should at least apply their methodology to DAIC-WOZ to prove the pipeline's validity.

Fix Data Inconsistencies: Resolve the participant count discrepancy (Abstract vs. Results).

Compare Features: In the Discussion, compare the specific significant features found (e.g., AU12, MFCCs) with those reported in Jiang et al. (2021) and Jiang et al. (2024) to discuss biological plausibility. In the Results, a breakdown of which features were most predictive and how many were used to achieve the highest performances is needed. Also, there are hyperparameters that were selected outside of the CV training, and the method of selecting needs to be defined/addressed. For instance, in the pre-processing, how were the parameters like 0.7 (line#163) selected?

Refine Labeling Discussion: Devote more discussion to the limitations of predicting a fluctuating symptom (SI) from a baseline static interview using time-series data.

Provide more than just AUC to compare: When comparing with the performances of others in the discussion, more info about the alternative works would be desired than just the AUC. At least the number of samples should be provided, and if they were a classification of the same/similar population?

More balance between claims and evidence: There are major claims that do not align with the evidence presented in the work. Particularly in the Discussion, it would be advisable to rewrite the claims that can be supported by the paper. A small study with a 7-day trial and identifying at-risk individuals of the MDD population in long-term real-life situations are not the same! Some examples of claims that need to be reevaluated can be seen in lines #(323,324) (338,339) Also (399,400)

5. Proofreading:

-Correct typos like "Appemdix" and ensure "P-values of the AUC" are calculated using appropriate tests for correlated ROC curves (e.g., DeLong).

-The Statistical analysis section is not very clear. For instance, what are the 3 groups mentioned (line#235)

-Line#138, Not clear how it mitigates data leakage?

-Lines#155,156 talk about moods and activities, but then talk about moods and not activities. Are the activities part dropped as a whole? If so, why?

-Through the paper, the discussion is about balanced data, but in lines 215,216 the imbalanced datasets are brought up, which makes it unnecessarily confusing.

Reviewer #3: Suicide prevention remains a major global health priority, and the integration of ecological momentary assessment (EMA) with machine learning represents a promising direction for scalable digital mental health solutions.

The manuscript is clearly written, ethically conducted, and aligned with the journal’s scope. The multimodal framework incorporating facial, acoustic, linguistic, and questionnaire-derived features collected via smartphone-based EMA is innovative and demonstrates feasibility in real-world settings. However, several methodological and translational issues limit the strength of the conclusions at this stage. I outline major and minor concerns below.

Major Concerns

1. Sample Size and High-Dimensional Feature Space

The study includes 99 participants (38 SI, 61 non-SI) with 1009 derived features. This results in a very high feature-to-sample ratio, raising substantial concerns regarding overfitting and model stability. Although ANOVA-based feature selection was performed within cross-validation folds, the dimensionality remains extremely large relative to sample size.

Please clarify:

Whether hyperparameter tuning and feature selection were fully nested within cross-validation.

Whether any stability analysis of selected features was performed across folds.

Whether confidence intervals for AUC and F1-score were calculated.

Without nested validation and stability assessment, performance estimates may be optimistic.

2. Absence of External Validation

All performance metrics are based on internal cross-validation. No external validation cohort was used. Given:

Single-site recruitment (Hong Kong),

Cantonese linguistic processing,

Cultural speech and affective expression patterns,

generalizability is uncertain.

The manuscript appropriately acknowledges this limitation, but the conclusions should be further tempered to emphasize the exploratory/proof-of-concept nature of the findings.

3. Binary Operationalization of Suicidal Ideation

Suicidal ideation was defined as H11 ≥ 1, collapsing passive death wishes, active ideation, and attempts into a single binary category. This may obscure clinically meaningful distinctions and introduces heterogeneity in the positive class.

Consider discussing:

Why ordinal or multiclass modeling was not attempted.

The distribution of H11 severity levels within the SI group.

Whether performance differs for higher-severity SI.

4. Incremental Value of Multimodal Features

HADS-A, HADS-D, and TAS subscales show strong group differences. It is unclear how much predictive value multimodal digital features add beyond questionnaire measures.

An ablation analysis would substantially strengthen the manuscript:

Questionnaire-only model

Multimodal digital features only

Combined model

This would clarify whether digital phenotyping provides meaningful incremental utility.

5. Clinical Utility and Sensitivity

The best-performing model (CatBoost) shows:

AUC = 0.79

Sensitivity = 0.53

Specificity = 0.89

While specificity is strong, sensitivity is modest, meaning nearly half of SI cases would be missed. For suicide screening, high sensitivity is typically prioritized.

Please discuss:

Intended clinical deployment scenario (screening vs. rule-in tool)

Threshold optimization strategy

Whether recall-focused optimization was considered

6. Model Interpretability

Interpretability is essential in psychiatric AI. The manuscript would benefit from:

Feature importance analysis

SHAP or similar explainability methods

Discussion of clinically interpretable signals

Understanding which modalities contribute most would improve translational relevance.

7. Data Availability and PLOS Policy

The Data Availability Statement indicates that data are available upon reasonable request. PLOS requires that all data underlying the findings be fully available without restriction, except in rare justified circumstances.

If privacy restrictions prevent full public release, please:

Specify the exact nature of restrictions,

Indicate whether de-identified feature matrices (without raw video/audio) can be deposited,

Clarify repository plans.

This is important for reproducibility.

Minor Comments

Some grammatical refinements are needed (“This study is a few of its kind”).

Claims such as “great capability” should be softened.

Consider adding calibration curves and reporting 95% confidence intervals for AUC.

Clarify the rationale for generating 980 derived features and whether dimensionality reduction alternatives were considered.

Overall Assessment

This study presents a promising digital mental health framework and addresses an important clinical problem. However, due to concerns regarding sample size, high dimensionality, absence of external validation, and limited interpretability analysis, the manuscript would benefit from substantial revision before it can be considered for publication.

With methodological clarification, additional analyses (especially ablation and calibration), and strengthened transparency regarding validation procedures and data sharing, this work could make a meaningful contribution to the field.

**********

what does this mean?). If published, this will include your full peer review and any attached files.

**Do you want your identity to be public for this peer review?**  If you choose “no”, your identity will remain anonymous but your review may still be made public.

For information about this choice, including consent withdrawal, please see our Privacy Policy

Reviewer #1: **Yes:**  Andreas M. Baumer

Reviewer #2: No

Reviewer #3: **Yes:**  Rafsan Jany

**********

**Figure resubmission:**

**Reproducibility:**

To enhance the reproducibility of your results, we recommend that authors of applicable studies deposit laboratory protocols in protocols.io, where a protocol can be assigned its own identifier (DOI) such that it can be cited independently in the future. Additionally, PLOS ONE offers an option to publish peer-reviewed clinical study protocols. Read more information on sharing protocols at https://plos.org/protocols?utm_medium=editorial-email&utm_source=authorletters&utm_campaign=protocols

# Author comment: Author response to Decision Letter 1

**DOI:** 10.1371/journal.pdig.0001615.r002

28 Apr 2026

Attachment Submitted filename: Response to Reviewers.docx

# Peer review: Decision Letter 1

**DOI:** 10.1371/journal.pdig.0001615.r003

**Authors:** Henry Horng-Shing Lu, Xiaoxia Wang

24 Jun 2026

Response to Reviewers Revised Manuscript with Track Changes Manuscript

**Journal Requirements:**

1. We note that you have included your Figures within the body of your manuscript. Please remove the Figures from the body of your manuscript and upload them as separate Figure files in .tif or .eps format.

For more information about figure files please see our guidelines:

https://journals.plos.org/digitalhealth/s/figures

https://journals.plos.org/digitalhealth/s/figures#loc-file-requirements

2. We have noticed that you have uploaded Supporting Information files, but you have not included a list of legends. Please add a full list of legends for your Supporting Information files after the references list.

**Additional Editor Comments (if provided):** **Reviewers' Comments:**

**Comments to the Author**

Reviewer #1: All comments have been addressed

Reviewer #3: All comments have been addressed

**********

publication criteria?

Reviewer #1: Yes

Reviewer #3: Partly

**********

3. Has the statistical analysis been performed appropriately and rigorously?

Reviewer #1: Yes

Reviewer #3: No

**********

4. Have the authors made all data underlying the findings in their manuscript fully available (please refer to the Data Availability Statement at the start of the manuscript PDF file)?

The PLOS Data policy

Reviewer #1: No

Reviewer #3: No

**********

5. Is the manuscript presented in an intelligible fashion and written in standard English?

Reviewer #1: Yes

Reviewer #3: Yes

**********

**Reviewer #1** : I thank the authors for their efforts in revising their manuscript. My comments have been sufficiently addressed.

**Reviewer #3:**  The revised manuscript is substantially improved compared with the previous version, particularly in the clarification of the study objectives, the discussion of limitations, the addition of baseline comparisons, and the expanded interpretation of the multimodal features.

The topic is clinically important, and the use of smartphone-based ecological momentary assessment combined with facial, acoustic, linguistic, and questionnaire-derived features represents an interesting and potentially useful direction for digital mental health research. I also appreciate the authors' efforts to frame the findings more cautiously as exploratory rather than presenting them as evidence of clinical readiness.

That said, several limitations remain and should continue to be emphasized throughout the manuscript.

First, the study is based on a relatively small cohort (N=99) with a high-dimensional feature space (>1000 features). Although feature selection was performed within the cross-validation framework, the risk of overfitting remains substantial. The absence of nested cross-validation and external validation makes it difficult to determine how well the reported performance would generalize to independent populations.

Second, while the newly added baseline comparison is valuable, the improvement achieved by incorporating multimodal features appears modest. This finding is still important, but it suggests that the multimodal signals should be interpreted as providing incremental rather than transformative predictive value.

Third, the manuscript would benefit from additional discussion regarding the stability and reproducibility of the selected features across folds. Given the exploratory nature of the work, understanding whether similar features consistently emerge would strengthen confidence in the reported digital biomarkers.

Finally, the lack of external validation remains the principal limitation of the study. I agree with the authors that publicly available datasets may not perfectly match the study design, language, or target population. Nevertheless, future validation in independent cohorts will be essential before any claims regarding clinical utility or deployment can be supported.

Overall, I believe the manuscript makes a useful contribution to the growing literature on multimodal digital assessment of suicidal ideation. The work should be interpreted as an exploratory proof-of-concept study demonstrating that multimodal EMA-derived features contain potentially meaningful information associated with clinician-rated suicidal ideation. Further validation in larger and independent cohorts will be required before conclusions regarding clinical applicability can be established.

**********

what does this mean?). If published, this will include your full peer review and any attached files.

**Do you want your identity to be public for this peer review?**  If you choose “no”, your identity will remain anonymous but your review may still be made public.

For information about this choice, including consent withdrawal, please see our Privacy Policy

Reviewer #1: No

Reviewer #3: **Yes:**  Rafsan Jany

**********

**Figure resubmission:**

**Reproducibility:**

To enhance the reproducibility of your results, we recommend that authors of applicable studies deposit laboratory protocols in protocols.io, where a protocol can be assigned its own identifier (DOI) such that it can be cited independently in the future. Additionally, PLOS ONE offers an option to publish peer-reviewed clinical study protocols. Read more information on sharing protocols at https://plos.org/protocols?utm_medium=editorial-email&utm_source=authorletters&utm_campaign=protocols

# Author comment: Author response to Decision Letter 2

**DOI:** 10.1371/journal.pdig.0001615.r004

30 Jun 2026

Attachment Submitted filename: Response to Reviewers_Marco_v5.1.docx

# Peer review: Decision Letter 2

**DOI:** 10.1371/journal.pdig.0001615.r005

**Authors:** Henry Horng-Shing Lu, Xiaoxia Wang

12 Jul 2026

Response to ReviewersRevised Manuscript with Track ChangesManuscript'.

**Journal Requirements:**

**Additional Editor Comments (if provided):**

**Reviewers' Comments:**

**Figure resubmission:**

**Reproducibility:**

-->To enhance the reproducibility of your results, we recommend that authors of applicable studies deposit laboratory protocols in protocols.io, where a protocol can be assigned its own identifier (DOI) such that it can be cited independently in the future. Additionally, PLOS ONE offers an option to publish peer-reviewed clinical study protocols. Read more information on sharing protocols at https://plos.org/protocols?utm_medium=editorial-email&utm_source=authorletters&utm_campaign=protocols
